# Structural and functional organization of the midline and intralaminar nuclei of the thalamus

**DOI:** 10.3389/fnbeh.2022.964644

**Published:** 2022-08-23

**Authors:** Robert P. Vertes, Stephanie B. Linley, Amanda K. P. Rojas

**Affiliations:** ^1^Center for Complex Systems and Brain Sciences, Florida Atlantic University, Boca Raton, FL, United States; ^2^Department of Psychology, Florida Atlantic University, Boca Raton, FL, United States; ^3^Department of Psychological Science, University of North Georgia, Dahlonega, GA, United States

**Keywords:** limbic thalamus, hippocampus, medial prefrontal cortex, striatum, arousal, cognition, affect

## Abstract

The midline and intralaminar nuclei of the thalamus form a major part of the “limbic thalamus;” that is, thalamic structures anatomically and functionally linked with the limbic forebrain. The midline nuclei consist of the paraventricular (PV) and paratenial nuclei, dorsally and the rhomboid and nucleus reuniens (RE), ventrally. The rostral intralaminar nuclei (ILt) consist of the central medial (CM), paracentral (PC) and central lateral (CL) nuclei. We presently concentrate on RE, PV, CM and CL nuclei of the thalamus. The nucleus reuniens receives a diverse array of input from limbic-related sites, and predominantly projects to the hippocampus and to “limbic” cortices. The RE participates in various cognitive functions including spatial working memory, executive functions (attention, behavioral flexibility) and affect/fear behavior. The PV receives significant limbic-related afferents, particularly the hypothalamus, and mainly distributes to “affective” structures of the forebrain including the bed nucleus of stria terminalis, nucleus accumbens and the amygdala. Accordingly, PV serves a critical role in “motivated behaviors” such as arousal, feeding/consummatory behavior and drug addiction. The rostral ILt receives both limbic and sensorimotor-related input and distributes widely over limbic and motor regions of the frontal cortex—and throughout the dorsal striatum. The intralaminar thalamus is critical for maintaining consciousness and directly participates in various sensorimotor functions (visuospatial or reaction time tasks) and cognitive tasks involving striatal-cortical interactions. As discussed herein, while each of the midline and intralaminar nuclei are anatomically and functionally distinct, they collectively serve a vital role in several affective, cognitive and executive behaviors – as major components of a brainstem-diencephalic-thalamocortical circuitry.

## Introduction

As well recognized, the thalamus is the gateway for the transfer of modality-specific information from principal (or first order) thalamic nuclei to distinct (sensorimotor) cortical targets. In the same manner, the thalamus is a conduit for the transfer of affective and cognitive-related (or limbic) information to distinct regions of the cortex which process this type of information—or limbic cortices. Accordingly, the thalamic nuclei which serve this function are generally recognized as constituting the “limbic thalamus”. Whereas, schemes may differ, the “limbic thalamus” is thought to mainly consist of the anterior nuclei (ATN), the mediodorsal nucleus (MD), the submedial nucleus, the intralaminar nuclei (ILt) and the midline nuclei (Vertes et al., 2015a,b). In this review, we describe: (1) the general organization of the thalamus; (2) the circuitry and functional properties of the midline and rostral intralaminar nuclei of the thalamus; and (3) the common and differential contribution of these thalamic groups to affective and cognitive behaviors.

### Organization of the thalamus

The thalamus has traditionally been divided into three anatomical/functional groups: the principal (or relay) nuclei, the association nuclei, and the midline and intralaminar nuclei (Jones, 1985, 2007; Vertes et al., 2015a,b). The principal “or relay” nuclei receive sensory or motor information through ascending pathways and transmit it to distinct regions of the cortex. The relay nuclei would include: the lateral geniculate complex (LGN), medial geniculate nucleus (MGN), ventral posteromedial (VPM) and posterolateral (VPL) nuclei, posterior nucleus (PO), ventral lateral nucleus (VL), ventral anterior nucleus (VA) and ventral medial nucleus (VM).

The “association” nuclei are a largely ill-defined group that differ from the principal nuclei in that they do not receive direct sensory (e.g., from the retina) or motor information and essentially do not project to primary sensorimotor cortices. The association nuclei receive major input from layer 5 pyramidal cells of the sensorimotor cortex and relay this information to associational areas of cortex—hence association nuclei of thalamus. The association thalamic nuclei include MD, the anterior nuclei, the submedial nucleus (SMT), and the lateral nuclei (lateral dorsal and lateral posterior).

The midline and intralaminar thalamic nuclei form a separate group primarily based on: (1) their distinct location along the midline and within the internal medullary lamina; and (2) and their relatively widespread distribution throughout the cortex. The intralaminar (ILt) nuclei consist of the central medial (CM), paracentral (PC), central lateral (CL), of the rostral ILt and the parafascicular (PF) and subparafascicular (SPF) nuclei of the posterior ILt. The midline nuclei include the paratenial nucleus (PT), paraventricular nucleus (PV), rhomboid nucleus (RH) and the nucleus reuniens (RE)—and in some classifications the intermediodorsal (IMD) nucleus.

### Midline and rostral intralaminar nuclei

**Figure 1:**
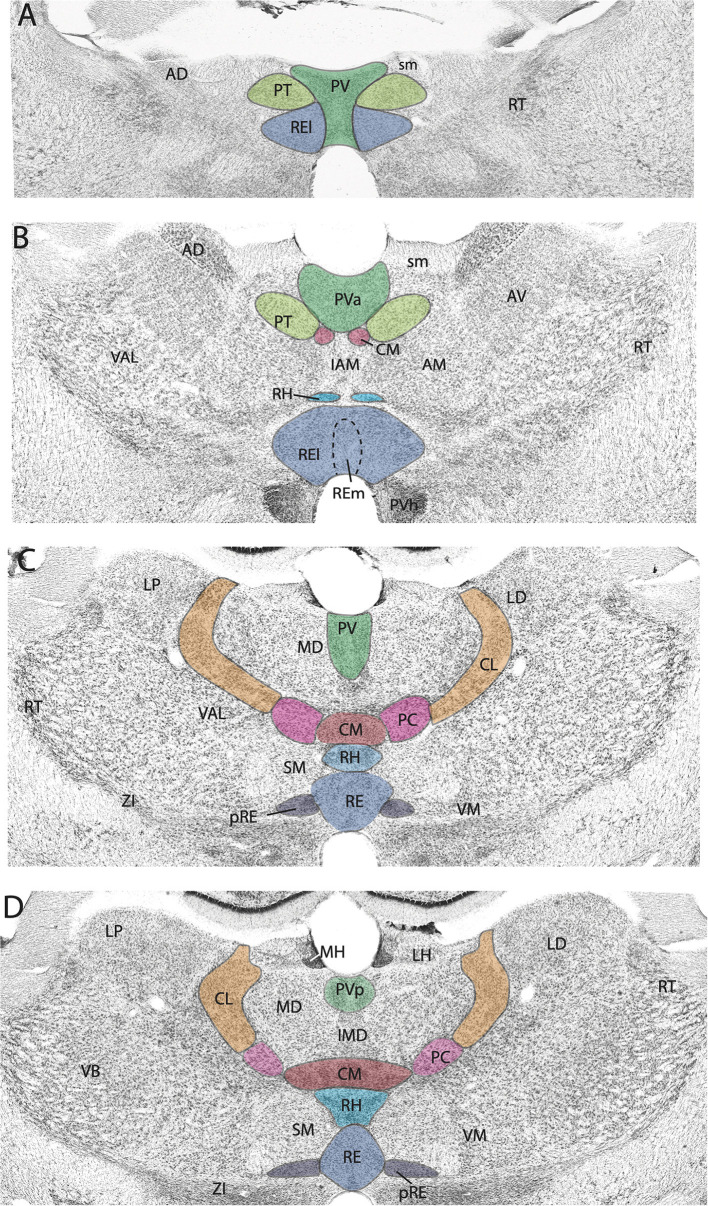
**(A–D)** Nissl-stained micrographs of transverse sections through the diencephalon of the rat depicting nuclei of the thalamus at four anterior to mid-levels of the thalamus. Colored-coded sections show the locations of the midline and rostral intralaminar nuclei of the thalamus. The midline nuclei consist of the paraventricular (PV) and paratenial (PT) of the dorsal midline thalamus and the rhomboid (RH) and reuniens (RE) nuclei of the ventral midline thalamus. The rostral intralaminar nuclei consists of the central medial (CM), paracentral (PC), and central lateral (CL) nuclei. AD, anterodorsal nucleus of thalamus; AM, anteromedial nucleus of thalamus; AV, anteroventral nucleus of thalamus; IAM, interanteromedial nucleus of thalamus; IMD, interomediodorsal nucleus of thalamus; LD, laterodorsal nucleus of thalamus; LH, lateral habenula; LP, lateral posterior nucleus of thalamus; MD, mediodorsal nucleus of thalamus; MH, medial habenula; pRE, peri-reuniens; PVa, anterior PV of thalamus; PVh, paraventricular nucleus of hypothalamus; PVp, posterior PV of thalamus; RE, nucleus reuniens, REl, REm, lateral, medial division of RE; RT, reticular nucleus of thalamus; sm, stria medullaris; SM, submedial nucleus of thalamus; VAL, ventral anterior nucleus of thalamus; VB, ventrobasal nucleus of thalamus; VM, ventromedial nucleus of thalamus; ZI, zona incerta.

The midline nuclei are characteristically divided into two main groups along the dorsoventral axis: the dorsal midline nuclei consisting of the paraventricular (PV) and paratenial (PT) nuclei and the ventral midline nuclei consisting of the rhomboid (RH) and reuniens (RE) nuclei. Presently, we focus on the circuitry and functional properties of RE and PV of the midline thalamus and the central medial nucleus (CM) of the rostral intralaminar complex in rodents (Figure 1).

## The ventral midline thalamus: Reuniens and rhomboid nuclei

As stated, the ventral midline thalamus consists of the RE and RH. While RE and RH projections are similar/overlapping, few studies have examined the functional properties of RH independent of RE. As such, we focus on RE. We first describe the circuitry of RE and then its functional properties.

### RE circuitry

#### RE input

RE receives a vast and diverse array of afferent projections from the cortex, hippocampus, basal forebrain, amygdala, hypothalamus and brainstem (Vertes, 2002, 2004; McKenna and Vertes, 2004; Hoover and Vertes, 2011; Scheel et al., 2020). Specifically, using retrograde tracers, McKenna and Vertes (2004) showed that RE receives widespread projections from subcortical and cortical sites. The main sources of cortical afferents to RE were from the orbitomedial prefrontal, insular, ectorhinal, perirhinal and retrosplenial cortices and the subiculum of the hippocampus (HF). The principal subcortical inputs were from the claustrum, lateral septum, bed nucleus of stria terminalis (BST), the medial, lateral, and magnocellular preoptic nuclei of the basal forebrain; the lateral habenula, PV and LGN of the thalamus; the zona incerta; the anterior, ventromedial, lateral, posterior, supramammillary and dorsal premammillary nuclei of the hypothalamus; and the ventral tegmental area (VTA), periaqueductal gray (PAG), precommissural nucleus, parabrachial nuclei, laterodorsal tegmental nucleus (LDT), and dorsal (DR) and median raphe (MR) nuclei of the brainstem.

In accord with findings in the rat (McKenna and Vertes, 2004), a recent examination of inputs to RE in the mouse (Scheel et al., 2020) similarly reported that RE receives a widely distributed set of afferents from subcortical and cortical sites. They described particularly dense projections to RE from deep layers of the HF and the medial prefrontal cortex (mPFC). Interestingly, no structures were found to project uniquely to RE in the mouse; that is, all structures projecting to RE in the mouse, also did so in the rat. On the other hand, several sites, including parts of the hypothalamus, BST and the amygdala were shown to distribute to RE in the rat (McKenna and Vertes, 2004) but not in the mouse (Scheel et al., 2020).

**Figure 2:**
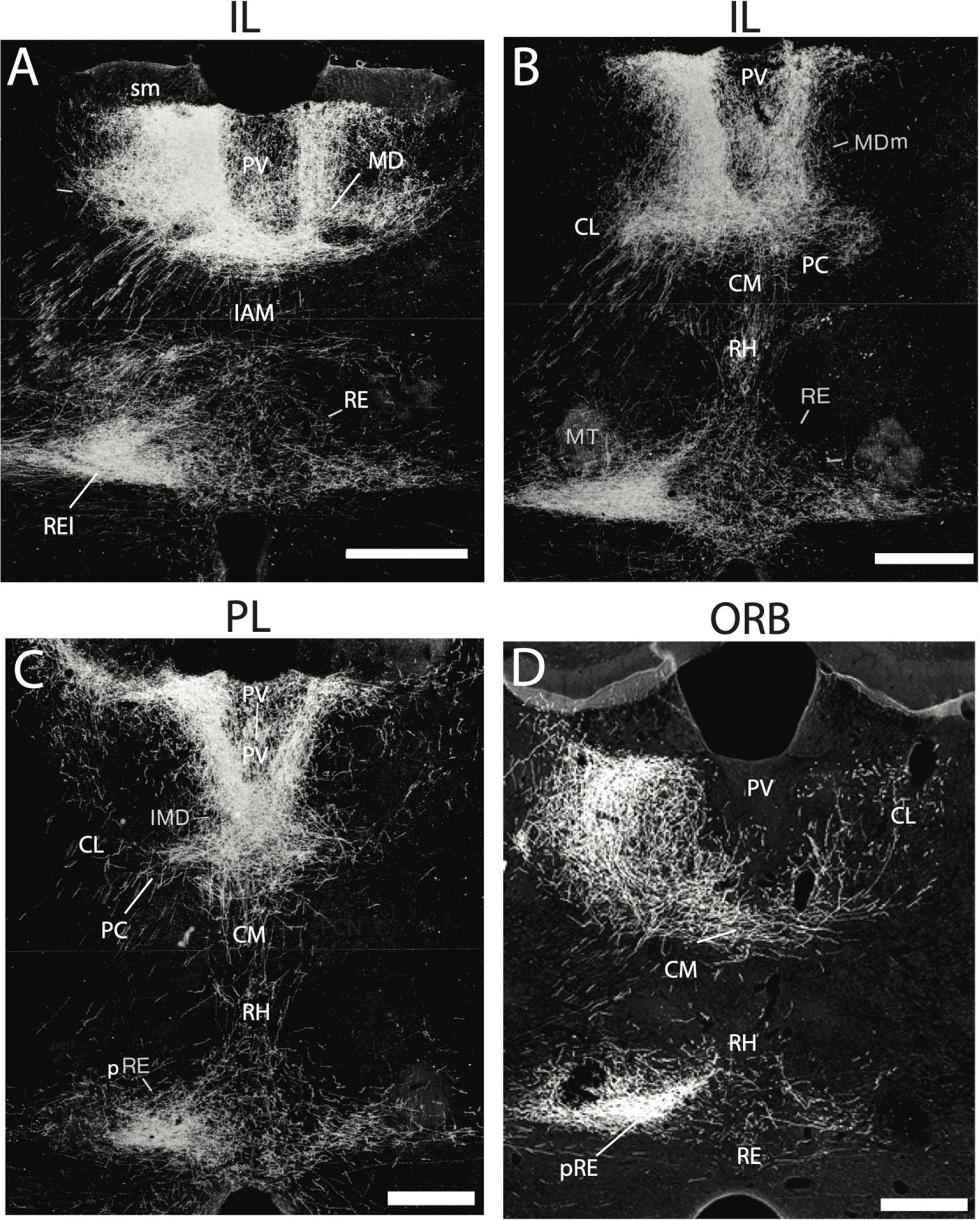
**(A–D)** Darkfield micrographs of transverse sections through the thalamus depicting patterns of anterograde labeling produced by PHA-L injection in the infralimbic (IL) **(A,B)** and prelimbic (PL) **(C)** cortices of the medial prefrontal cortex (mPFC) and the ventral orbital cortex (ORB) **(D)**. As depicted, injections in IL **(A,B)** and PL **(C)** gave rise dense terminal labeling of the paraventricular nucleus (PV) and medial division of mediodorsal nucleus (MDm), dorsally and rhomboid (RH) and the nucleus reuniens (RE), ventrally, with intense labeling of the lateral wings of RE (REl), rostrally **(A)** and peri-reuniens (pRE), caudally **(B,C)**. By comparison, injections in the ventral orbital cortex (ORB) **(D)** produced dense labeling of the central medial (CM), paracentral (PC) and central lateral (CL) nuclei of the rostral intralaminar complex, heaviest in CL, ipsilaterally (left side) as well as pronounced labeling of the nucleus reuniens – comparable to that seen with injections in IL and PL **(A–C)**. IAM, interanteromedial dorsal nucleus of thalamus; IMD, interomediodorsal nucleus of thalamus; MD, mediodorsal nucleus of thalamus; MT, mammillothalamic tract; sm, stria medullaris. Scale bar for **(A–D)** = 450 μm. Figure modified from Vertes (2002) and Hoover and Vertes (2011).

RE projects widely to limbic cortices, densely to the HF and mPFC, but also prominently to the orbital, insular, retrosplenial, and parahippocampal cortices (see below). With the possible exception of the entorhinal cortex, each of these cortical regions are sources of afferent (return) projections to RE (Figure 2), indicating strong reciprocal connections between RE and these cortical sites (Vertes, 2002, 2004; Hoover and Vertes, 2011). For instance, anterograde PHA-L injections in the infralimbic (IL) or prelimbic (PL) cortices of the mPFC were shown to produce massive terminal labeling throughout dorsoventral extent of the midline thalamus, most heavily in RE (Figures 2A–C) and the medial division of MD (MDm) (Vertes, 2002, 2004). Strikingly, there were (virtually) no IL/PL projections to lateral regions (or principal nuclei) of the thalamus. Moreover, mPFC projections to RE appear topographically organized such that IL/PL fibers distribute heavily to the lateral wings of RE or the peri-reuniens, (pRE) (Figures 2A–C). which in turn, is the main source of return projections to the mPFC (Vertes, 2002, 2004; Jayachandran et al., 2019). With respect to the hippocampus, injections of retrograde tracers into RE were found to produce a dense band of labeled cells throughout the length of the ventral subiculum of the mouse (Scheel et al., 2020) and the rat (McKenna and Vertes, 2004).

#### RE output

**Figure 3:**
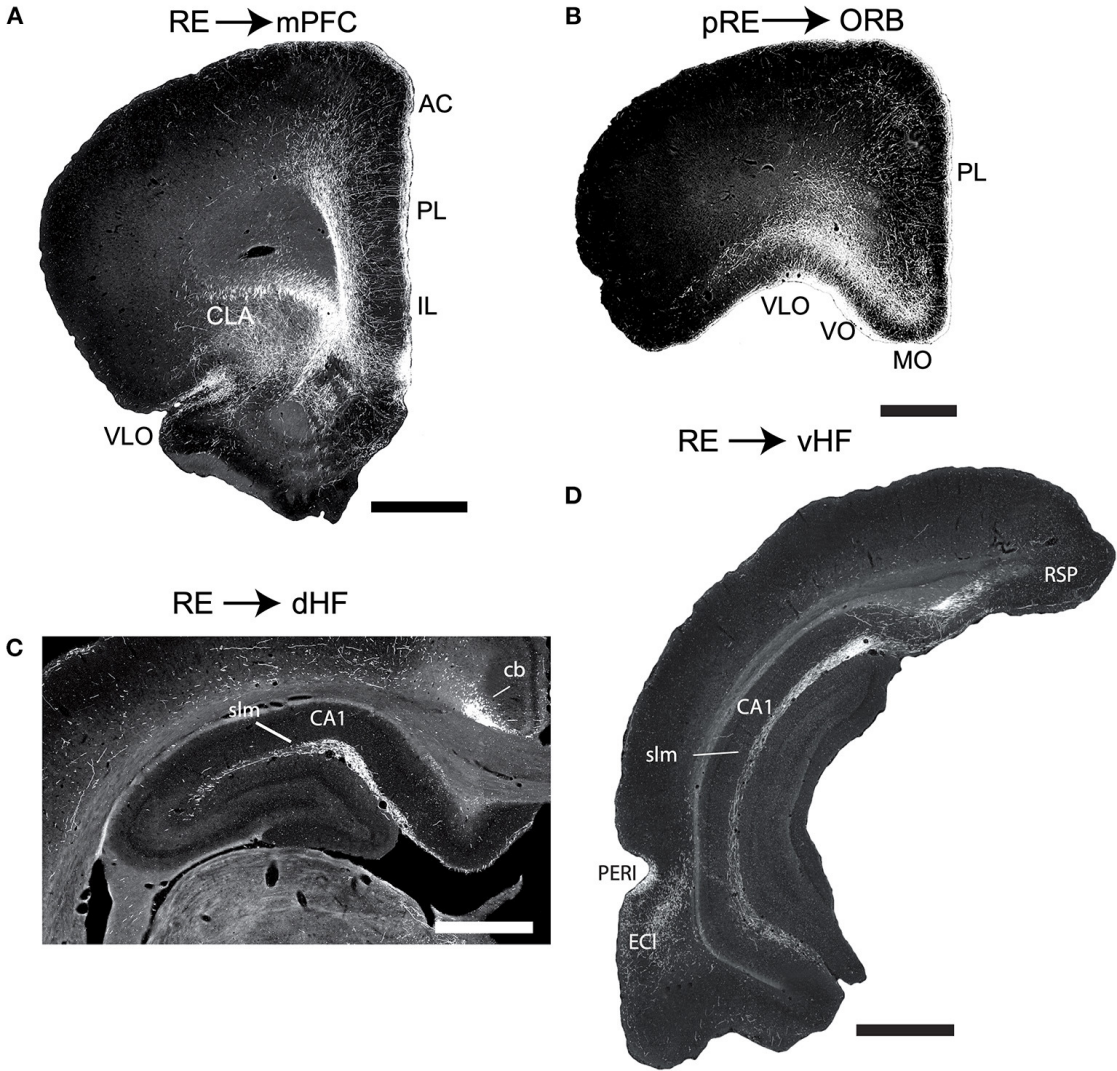
**(A,B)** Low magnification darkfield micrographs of transverse sections through the forebrain depicting the pattern of labeling in the medial prefrontal cortex (mPFC) **(A)** and the orbital cortex (ORB) **(B)** in the rat produced by anterograde tracer injections in the nucleus reuniens (RE) and peri-reuniens (pRE), respectively, of the ventral midline thalamus. **(A)** Note the dense collection of labeled fibers in the anterior cingulate (AC), prelimbic (PL) and infralimbic (IL) cortex of the mPFC, most concentrated in layers 1 and 5/6. **(B)** Note the dense labeling extending mediolaterally across ORB, heavily concentrated in the medial (MO) and ventral (VO) divisions of the ORB. **(C,D)** Low magnification darkfield micrographs of transverse sections through the dorsal **(C)** and ventral hippocampus **(D)** depicting patterns of labeling following anterograde tracer injections (PHA-L) in the nucleus reuniens. Note the dense collection of labeled fibers in the stratum lacunosum moleculare (slm) of CA1 of the dorsal **(C)** and ventral hippocampus and in molecular layer of the ventral subiculum (SUBv) **(D)**. Scale bar for **(A,D)** = 1000 μm; for **(B)** = 750 μm; for **(C)** = 600 μm. Abbreviations: cb, cingulum bundle; CLA, claustrum; DLO, dorsolateral orbital cortex; ENTl, lateral entorhinal cortex; PERI, perirhinal cortex; RSP, retrosplenial cortex; VLO, ventrolateral orbital cortex. Figure modified from Vertes et al. (2006).

The major efferent targets of RE are the hippocampus (HF) and limbic (neo) cortices. RE distributes prominently to the IL, PL, and anterior cingulate (AC) cortices of the mPFC (Figure 3A), but also significantly to the medial (MO) and ventral orbital (VO) cortices (Figure 3B), the dorsal (AId) and ventral agranular (AIv) insular cortices, the rostral retrosplenial cortex, the perirhinal cortex, and the medial and lateral entorhinal (EC) cortices. With the exception of projections to the rostral pole of nucleus accumbens (ACC), RE gives rise to limited projections to subcortical structures (Vertes et al., 2006).

As described in several reports, RE distributes massively, and in a highly organized manner, to the hippocampus. Specifically, RE fibers to the HF terminate selectively in the stratum lacunosum-moleculare (slm) of CA1 of the dorsal and ventral HF and in the molecular layer of the subiculum (SUB) and parasubiculum (Figures 3C,D). RE axons mainly form asymmetric (excitatory) contacts predominantly on distal dendrites of pyramidal cells in the slm of CA1 and SUB. There are no RE projections to CA2 and CA3 of Ammon's horn or to the dentate gyrus of the hippocampus (Herkenham, 1978; Wouterlood et al., 1990; Van der Werf et al., 2002; Vertes et al., 2006, 2007; Hoover and Vertes, 2012).

Recent reports using retrograde fluorescent tracers have described collateral RE projections to its two main targets, the HF and the mPFC (Hoover and Vertes, 2012; Varela et al., 2014). Specifically, Hoover and Vertes (2012) found that ~5–10% of RE cells distributed, *via* collaterals, to the HF and mPFC – mainly concentrated laterally in RE, just medial to the lateral wings of RE. Although RE cells projecting to single sites (i.e., non-branching) were intermingled throughout RE, those distributing to the mPFC were mainly located in the lateral wings of RE, while those projecting to the HF were most numerous in the rostral pole of RE. Interestingly, RE projections to the ventral HF were ~10-fold greater than those to the dorsal HF (Hoover and Vertes, 2012).

Varela et al. (2014) similarly showed that about 8% of RE cells, spanning its length, gave rise to collateral projections to the HF and the ventral mPFC. They further reported that only ~ 1% of subicular neurons projected *via* collaterals to RE and to the mPFC. It was suggested that RE cells with branching projections to HF and the mPFC may serve a role in memory consolidation through the synchronization of theta activity of these structures.

Whereas, the hippocampus projects strongly to the mPFC, interestingly, there are no direct return projections from the mPFC to the HF (Sesack et al., 1989; Laroche et al., 2000; Vertes, 2004). The demonstration that the mPFC strongly targets the RE, and reuniens in turn, distributes massively to the hippocampus indicates that RE is a main link from the mPFC to the hippocampus—thus completing a loop between these structures: HF > mPFC > RE > HF. Supporting this, it was demonstrated, at the ultrastructural level, that mPFC fibers distributing to RE form asymmetric (excitatory) contacts on proximal dendrites of RE cells projecting to the hippocampus (Vertes et al., 2007).

**Figure 4:**
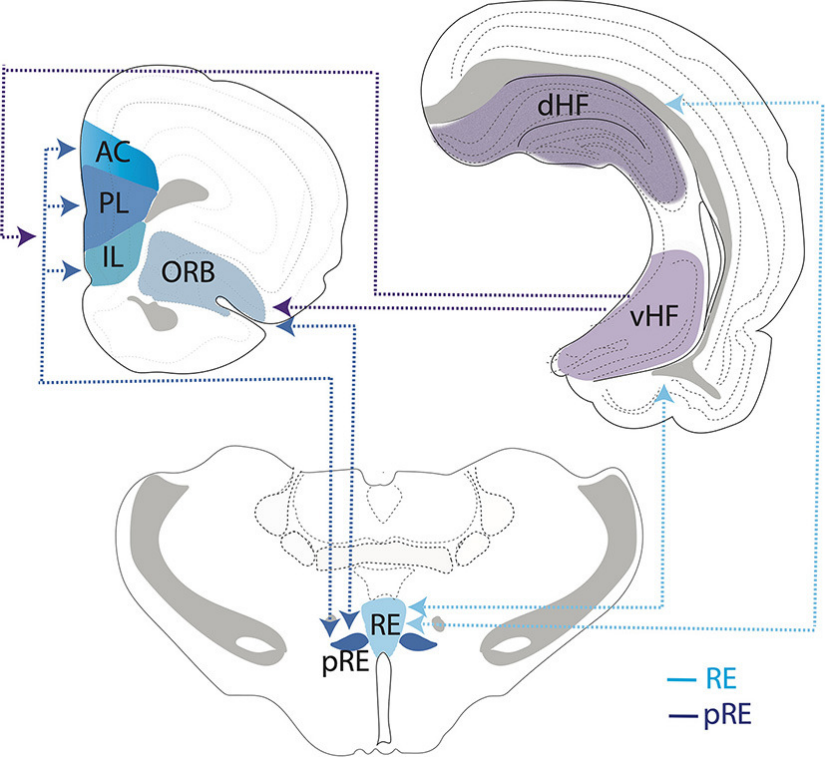
Schematic representation depicting interconnections/circuitry between the nucleus reuniens (RE) and the peri-reuniens (pRE), the medial (mPFC) and orbital (ORB) prefrontal cortices and the hippocampus (HF). While both RE and pRE are interconnected with the mPFC, ORB and HF, there is differential weighting of connections such that the RE (or medial RE) is more heavily reciprocally connected with the dorsal (dHF) and ventral (vHF) hippocampus (light blue lines/arrows), whereas pRE is more strongly reciprocally connected with the mPFC and ORB cortices (dark blue lines/arrows). While the vHF sends projections to the mPFC/ORB (purple lines/arrows), there are essentially no return projections from the orbitomedial PFC cortex to the HF. Further, the dHF does not project directly to the mPFC/ORB. As such, RE/pRE is a key intermediary in this circuitry. Dashed lines and arrows represent direction of connections. AC, anterior cingulate cortex; IL, infralimbic cortex; PL, prelimbic cortex.

In addition to RE, another prominent input to the HF is the lateral entorhinal cortex (ECl). In this regard, Schlecht et al. (2022) recently examined possible sources of dual projections to RE and ECl and described dually projecting cells in the medial septum and ventral subiculum but interestingly not in the mPFC—indicating that separate populations of mPFC cells project to the RE and ECl. Figure 4 summarizes the main interconnections of RE (and peri-reuniens, pRE) with the mPFC (IL, PL, AC), orbital cortex, and the dorsal and ventral hippocampus.

### Nucleus reuniens: Functional properties—Overview

RE is reciprocally connected with the HF and several limbic cortical sites, prominently the mPFC, and is a major intermediary between the HF and mPFC (Cassel et al., 2013, 2021; Griffin, 2015, 2021; Vertes et al., 2015a; Dolleman-van der Weel et al., 2019; Ferraris et al., 2021). Accordingly, RE has been associated with several, diverse affective and cognitive functions—or notably those involving interactions between the HF and orbitomedial PFC. These include working memory (WM)/spatial working memory (SWM), executive functions (attention, goal directed behavior, decision making) and affective/fear behavior.

### RE functional properties—WM/SWM

It is well recognized that the hippocampus and mPFC serve critical roles in SWM (Colgin, 2011; Griffin, 2015). Lesions or inactivation of either structure in rats produces severe deficits in SWM (Floresco et al., 1997; Lee and Kesner, 2003; Jones and Wilson, 2005; Yoon et al., 2008; Churchwell et al., 2010; Churchwell and Kesner, 2011; Hallock et al., 2013a; O'Neill et al., 2013; Urban et al., 2014; Sapiurka et al., 2016; Avigan et al., 2020). As an interface between HF and mPFC, RE is well positioned to coordinate their activity in SWM—and other tasks. As such, alterations of RE have been shown to disrupt HF-mPFC communication—leading to deficits on WM tasks (Hembrook and Mair, 2011; Hembrook et al., 2012; Cholvin et al., 2013; Hallock et al., 2013b, 2016; Duan et al., 2015; Layfield et al., 2015; Maisson et al., 2018; Viena et al., 2018).

For instance, Mair et al. (Hembrook and Mair, 2011; Hembrook et al., 2012) showed that lesions of RE (and the dorsally adjacent, RH) significantly altered performance on WM tasks that are sensitive to damage to the HF or the mPFC. RE lesions, however, had no effect on tasks involving alterations of the striatum and motor cortex such as visuospatial tasks, or interestingly those which only involved the hippocampus such as certain radial arm maze (RAM) tasks. Regarding the latter, Hembrook et al. (2012) concluded that “the RE and RH affect measures of spatial working memory that depend on interactions between the hippocampus and mPFC, but not measures that depend on the hippocampus alone”.

Griffin et al. (Hallock et al., 2013b, 2016) similarly reported that alterations of RE disrupt WM behaviors dependent on HF-mPFC interactions and further described RE-mediated synchronous oscillations between the HF and mPFC supporting WM behavior. Specifically, Hallock et al. (2013b) initially examined the effects of inactivation of RE on two versions of tactile/visual T-maze task, one with and the other without, a WM component, and showed that RE rats only exhibited deficits on the WM version of the task. In a subsequent examination of HF-mPFC unit/oscillatory activity during WM behavior, Hallock et al. (2016) showed that: (1) a population of mPFC cells, active during a delayed SWM task, became entrained to hippocampal theta during successful task performance; (2) hippocampal theta was strongly coupled to theta and gamma oscillations of the mPFC under the same conditions; and (3) the reversible inactivation of RE with muscimol disrupted HF-mPFC synchronous oscillations as well as performance on the delayed SWM task.

**Figure 5:**
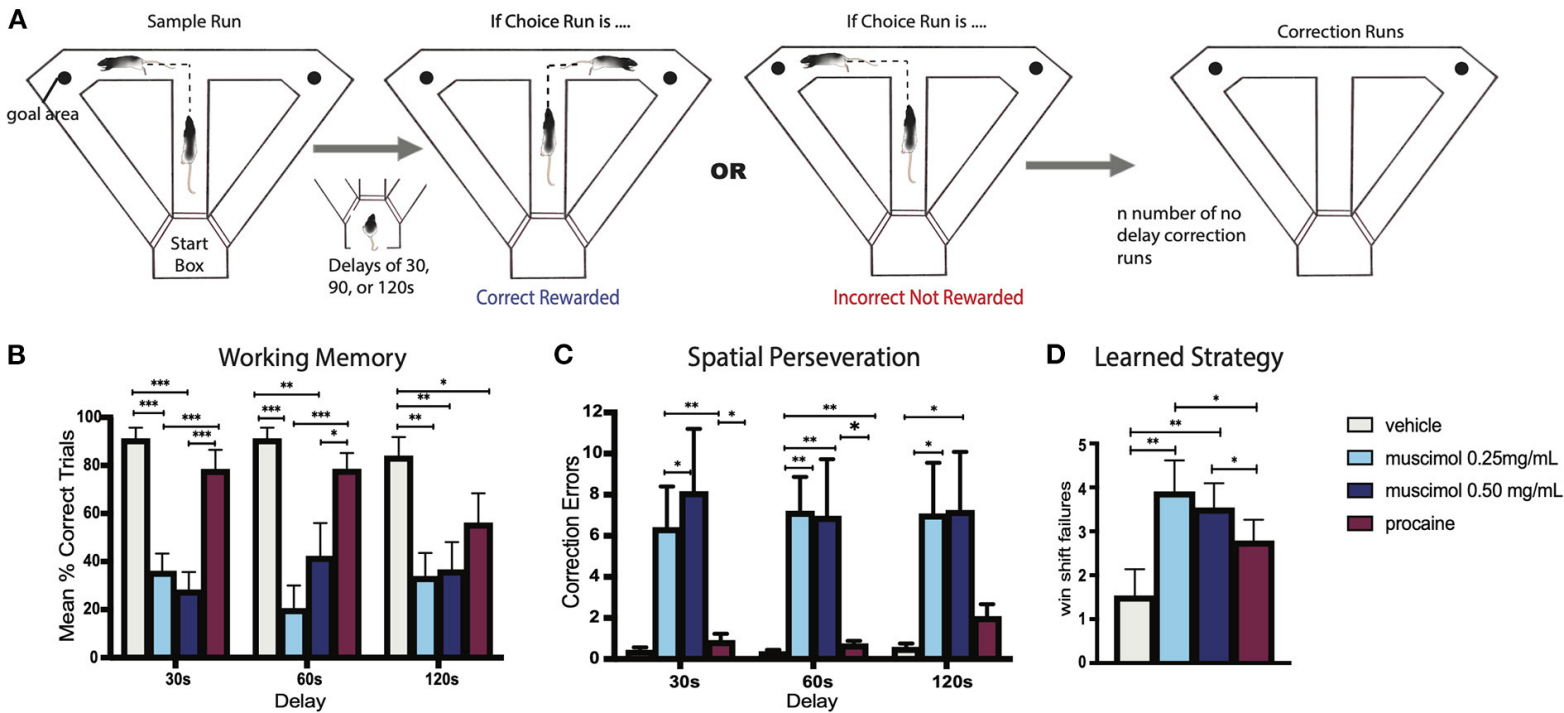
**(A)** Experimental design of a modified delayed non-match to sample (DNMS) T-maze task used to examine spatial working memory and behavioral flexibility in the rat. Rats began each trial with a free choice on the sample run, whereby they could choose either arm for reward. Following this, rats returned to the startbox and remained there for delays of 30, 60, or 90 s before the start of the choice run whereby the correct choice involved choosing the opposite arm. If rats made an incorrect response on the choice run, they were given no delay correction runs which allowed them to immediately correct their error and choose the correct arm. If a rat did not correct their behavior after 10 “correction” runs, the trial was terminated and rats were returned to the startbox. This was followed by the next trial. **(B)** Bar graph illustrating spatial working memory performance on the DNMS task following infusions of muscimol, procaine, or vehicle into the nucleus reuniens (RE). Infusions of muscimol, at two doses, into RE impaired choice accuracy at each of the three delay times, measured by mean percent of correct trials, demonstrating that the inactivation of RE profoundly disrupts spatial working memory. By contrast, procaine injections in RE impaired choice accuracy only during the longest delay (120 s). **(C)** Bar graph showing errors made during correction runs following infusions into RE. Inactivation of RE with muscimol at two doses produced striking spatial perseverative behavior on the DNMS task whereby rats repeatedly reentered the incorrect arm on correction runs, despite the absence of reward. **(D)** Bar graph of win-shift failures made across testing sessions following infusions into RE. Rats well-trained in the DNMS task learned to alternate with choice runs, which included alternating on the sample run following the choice run on the previous trial. Muscimol infusions into RE disrupted this behavioral strategy, significantly increasing the number of win-shift errors, by which rats did not alternate across trials. Error bars represent standard error of the mean. Significance is indicated by asterisks: *p < 0.05; **p < 0.01; ***p < 0.001. Modified from Viena et al. (2018).

Viena et al. (2018) assessed the role of RE in SWM using a variant of the delayed non-match to sample (DNMS) T-maze task wherein rats were allowed to correct their behavior following incorrect choices on the T-maze (Figure 5A). Specifically, rats were given a free choice of the right or left arm of the T-maze (sample run) and after delays of 30, 60 or 120 s were required to choose the opposite arm of the maze (choice run) for reward. If rats chose the incorrect arm on the choice run, they were allowed to immediately (without delay) correct their behavior by choosing the correct (or baited) arm. The repeated re-entry into the incorrect (non-baited) arm was defined as a perseverative error. The reversible inactivation of RE across two doses of muscimol severely disrupted performance on this task, impairing choice accuracy at each of the three delay times (Figure 5B). In addition, muscimol injections into RE resulted in a pronounced spatial perseveration (Figure 5C), as rats repeatedly choose the incorrect arm in the absence of reward—or were unable to shift response strategies (see also below).

In a comparable manner, Griffin and colleagues (Stout et al., 2022) recently examined the role of RE in *deliberation*—or specifically in pause and reorienting behaviors which have been referred to as “Vicarious Trial and Errors” (VTEs) (Schmidt et al., 2013; Redish, 2016). Interestingly, VTEs appear to increase with unsuccessful deliberations in WM tasks (Redish, 2016). Further, as discussed, theta oscillations between the HF and mPFC become synchronized during successful choice behavior on WM tasks (Jones and Wilson, 2005; O'Neill et al., 2013). Accordingly, Stout et al. (2022) demonstrated that the inactivation of RE, increased VTEs, suppressed HF-mPFC theta synchrony and significantly impaired performance on a spatial WM task, resulting in perseverative errors.

Despite these studies, the precise role(s) of RE in spatial WM remains unclear; that is, is the involvement of RE in WM linked to the encoding, retrieval or long term retention of spatial information? Specifically, Maisson et al. (2018) using a delayed non-match to position (DNMP) T-maze task, showed that optogenetic inhibition of RE during the sample phase, but not during the delay or choice phases (retrieval) of the task, significantly impaired performance on the task, and concluded that RE mainly contributes to the encoding of spatial information in SWM tasks. Conversely, Rahman et al. (2021) recently demonstrated that optogenetically stimulating RE hippocampal-projecting fibers at delta frequency (3–4 Hz) in mice, which had previously been shown to disrupt memory processing in rats (Duan et al., 2015), significantly impaired the retrieval but not the encoding of memory on a spatial Y-maze task. Lastly, Jayachandran et al. (2019) described the involvement of RE in the temporal coding of working memory. They found that the chemogenetic silencing of mPFC projections to RE impaired memory for the sequential order of odor stimuli, and based on the pattern of deficits, concluded that altering mPFC-RE projections produces failures of a WM retrieval strategy. While these conflicting findings need to be resolved, it would appear from the foregoing that RE serves a role in various phases of spatial learning tasks, and disparities among studies could involve differences in types of tasks, perturbations of RE, timescales, species—or possibly other variables.

In summary, working memory critically involves interactions between the HF and the mPFC, and as recently demonstrated, RE represents a vital link between the HF and the mPFC in working memory. Accordingly, the inactivation of RE disrupts both synchronous oscillations between the HF and mPFC and performance on working memory tasks as well as long-term memory processing.

### RE functional properties—Executive functions

Whereas, RE's involvement in SWM has been extensively examined (for review, Cassel et al., 2013; Griffin, 2015; Vertes et al., 2015a; Dolleman-van der Weel et al., 2019), considerably fewer reports have assessed its role in behaviors that have been termed “executive functions.” They would include attentional processes, behavioral flexibility, decision making, and goal directed behavior (Dolleman-van der Weel et al., 2009; Cholvin et al., 2013; Prasad et al., 2013; Linley et al., 2016; Viena et al., 2018).

In an initial study, Dolleman-van der Weel et al. (2009), reported that RE lesions did not disrupt acquisition or retention on a standard water maze task, but nonetheless resulted in an ineffective search strategy on the probe test which was described as a rigid (or inflexible) behavioral pattern—or a prefrontal cortical rather than a hippocampal deficit. In a similar manner Cassel and colleagues (Cholvin et al., 2013) compared the effects of the selective inactivation of the HF, the mPFC or RE on a standard water maze and on a double-H water maze task that places demands on both the hippocampus (place identification) and the mPFC (strategy-shifting) for successful completion. Hippocampal, but not RE, inactivation impaired performance on the standard water maze, whereas the inactivation of RE, HF, or the mPFC disrupted performance on the double-H task, resulting in an inability to successfully switch strategies on the task. Specifically, the RE (inactivated) rats were unable to switch from an incorrect response strategy (repeating a learned sequence of movements) to a correct place response (choosing the escape quadrant)—indicating a deficit in behavioral flexibility.

Linley et al. (2016), using odor-tactile attentional set shifting task (AST), reported that RE lesioned rats were impaired on the ability to establish “attentional sets” and in reversal learning. The odor tactile AST task of Verity Brown (see Brown and Tait, 2015) consists of 7 stages requiring rats to dig for food rewards buried in various mediums of scented food cups. The seven stages are: simple discrimination (SD), compound discrimination (CD), intradimensional shift (ID), extradimensional (ED) shift, and reversal learning of the CD, ID, and ED stages. Linley et al. (2016) found that RE lesioned rats exhibited significant deficits on the intradimensional shift and first reversal (CDRV) stages of the AST task. This indicated: (1) an inability to learn successful rules (or strategies) that would transfer or generalize across a comparable set of tasks/stimuli; and (2) a failure to inhibit responses to previously rewarded stimuli to thereby initiate responding to once unrewarded stimuli—or inflexible behavior.

As was previously discussed, Viena et al. (2018) reported that muscimol injections in RE produced severe spatial “perseverative” behavior on a T-maze alternation task – wherein rats repeatedly made incorrect directional responses on the maze (Figure 5C). This perseverative (or compulsive) responding is a further example of the inability of RE rats to alter their behavior in the face of changing contingencies or conditions—an executive dysfunction. Further, muscimol inactivation also disrupted the ability of rats to use a well-established rule of spatial alternation across trials—signifying win-shift errors (Figure 5D). Together this demonstrated a pivotal role for RE in learned strategies and flexible behavior.

Spatial perseveration has been linked to dysregulation of the hippocampus. Dalland (1970, 1976) initially showed that lesions of the dorsal hippocampus produced spatial perseveration on a spatial alternation task. More recently, Hallock et al. (2013a) compared the effects of reversible inactivation of the dorsal HF or the striatum on a spatial (delayed alternation) or non-spatial (visual discrimination) task, and found that disruption of the HF, but not the striatum, impaired performance on the delayed alternation task, notably increasing scores on a “perseveration index,” which measured re-entries into previously visited arms. Comparably, Yoon et al. (2008) described the effects of reversible inactivation of the mPFC or the HF on delayed alternations in a Figure 8 maze showing that disruption of the mPFC increased WM errors, whereas altering the HF significantly increased perseverative errors. Finally, Zhang et al. (2013), using a DNMS task, reported that NMDA antagonists applied to CA1 significantly impaired the ability of rats: (1) to correct their behavior following errors, termed “lose-shift errors,” leading to spatial perseveration and (2) to execute a well-learned strategy and alternate following successful choices, or win-shift errors. As discussed, these same deficits (perseveration and win-shift errors, Figures 5B–D) were observed on the DNMS task following RE inactivation (Viena et al., 2018).

Whereas, executive functions undoubtedly involve a widely distributed cortical network (Dalley et al., 2004; Robbins and Arnsten, 2009; Kesner and Churchwell, 2011; Sharpe and Killcross, 2018), alterations of the orbital cortex (ORB) commonly result in inflexible behavior, while (as discussed) disruptions of the hippocampus appear to underlie spatial perseverative responding.

The association of the orbital corex (ORB) with behavioral flexibility is often examined in animal models through reversal learning or compulsive behavior (for review, Clark et al., 2004; Schoenbaum et al., 2009; Young and Shapiro, 2011a; Izquierdo, 2017). For instance, it has been shown that disruption of the ORB in rats impairs the intradimensional shift and reversal learning phases of the AST task (McAlonan and Brown, 2003; Chase et al., 2012). Further, several reports in rats have shown that ORB cells respond differentially to correct and incorrect (reward based) choices. Using a two-choice odor discrimination task, Feierstein et al. (2006) demonstrated that ORB neurons fired in response to outcome (reward vs. non-reward) and to correct choice locations. Steiner and Redish (2012) similarly reported that ORB cells in rats discharged selectively on rewarded trials of a maze, with activity peaking at the choice point of the maze. Finally, Young and Shapiro (2011b) showed that ORB activity was correlated with reward probabilities of paths taken on a plus maze, and further that theta of the HF and ORB became synchronized with successful performance on the task. The foregoing demonstrates a critical involvement of ORB in establishing reward-response contingencies and importantly for adapting to changes in contingencies (i.e., reversal learning)—as alterations of ORB severely disrupt reversal learning.

While the manner in which the ORB acquires the necessary information for evaluative decisions has yet to be been fully determined, Wikenheiser and Schoenbaum (2016) proposed that spatial and contextual features of the environment, encoded by the hippocampus, are relayed to the ORB—and there evaluated for reward/valence properties for appropriate behavioral responses. Further, the ORB would then transmit information on reward outcomes (or behavioral adaptations) from the ORB to the HF to update it, thus preparing the HF for future goal directed actions. This would obviously require a functional interplay between the HF and ORB, but the ORB does not receive (direct) input from the dorsal HF, and the ORB does not project to CA1 and the subiculum of the HF (Dolleman-van der Weel and Witter, 1996; Reep et al., 1996; Vertes et al., 2006, 2007; Hoover and Vertes, 2011, 2012; Prasad and Chudasama, 2013; Murphy and Deutch, 2018). As RE serves as a primary link between the mPFC and HF, RE is also reciprocally connected with the ORB (Van der Werf et al., 2002; Jasmin et al., 2004; McKenna and Vertes, 2004; Vertes et al., 2006; Hoover and Vertes, 2011) and thus may be a key intermediary in the exchange of information between the HF and the ORB (Figure 4). As such, deficits seen with the disruption of RE on tasks involving attention/attentional set, reversal learning and behavior flexibility (perseveration) may, in part, involve the loss of effective RE-mediated communication between the HF and ORB.

In summary, compared to reports on working memory, considerably fewer studies have examined the role of RE in behaviors designated as “executive functions” such as attentional set, behavioral flexibility, goal directed behavior and decision making. Recent evidence, however, indicates that alterations of RE significantly disrupt attentional processes and behavioral flexibility—primarily involving RE connections with the mPFC, ORB and the hippocampus.

### RE functional properties—Affect/fear behavior

It is well recognized that alterations of the HF, the mPFC or their interactions underlie several affective disorders including depression, anxiety, and post-traumatic stress disorder (PTSD) (Jin and Maren, 2015; Sigurdsson and Duvarci, 2016). As RE is a major link between the HF and the mPFC, RE appears to serve a critical role in affective behavior.

In this regard, several reports have described a direct involvement of RE in emotional behavior, most thoroughly examined with respect to fear—using fear conditioning paradigms. For instance, Xu and Südhof (2013) described a mPFC-RE-HF circuit responsible for fear memory specificity and generalization. They initially showed that alterations of the mPFC produced to an overgeneralization of fear memory (Xu et al., 2012), and subsequently that this effect was dependent on mPFC actions on the HF, mediated by RE. Specifically, they demonstrated that disruption of mPFC projections to RE, but not to other thalamic sites, produced an overgeneralization of contextual fear memory, and that the suppression or enhancement of RE output to the HF, heightened or reduced overgeneralized contextual fear memory, respectively (Xu and Südhof, 2013).

Wheeler et al. (2013) examined patterns of c-fos expression across 84 regions of the brain following the recall of contextual fear memory in mice, and described specific regions of the brain which were co-activated by fear recall, leading to the identification of “hubs” in a fear network—or highly interconnected structures of the network. Having identified 4 of 21 regions of the brain as “hubs” (CA1 of HF, RE, lateral septum and laterodorsal nucleus of thalamus), they showed that chemogenetic silencing of these hubs significantly impaired fear memory consolidation (Vetere et al., 2017). They concluded that: “hubs play disproportionately important roles, in a network engaged by contextual fear memory in mice”.

Sierra et al. (2017) showed that remote contextual fear memory (blocked by suppressing the cortex during conditioning) could be reinstated by “reconsolidation” which was dependent on RE; that is, inactivating RE prevented the reinstatement of remote fear memory. They proposed that (fear) contextual information was conveyed from the HF to the PFC, *via* RE, to consolidate fear/emotional memories in the PFC. Consistent with this, Quet et al. (2020) recently demonstrated that RE/RH lesions in rats significantly disrupted remote (25 days), but not recent (1 day), contextual fear memory. Further, RE/RH was not required for the retrieval of remote fear memory, thus restricting its involvement to the consolidation of remote fear memories.

In a similar manner, Ramanathan et al. (2018a) showed that the inactivation of RE severely disrupted the acquisition and expression of contextual fear memory, and interestingly it also released (or uncovered) an elemental, non-hippocampal dependent contextual memory system—producing an overgeneralization of contextual fear to novel contexts. They thus concluded that RE encodes precise HF-dependent contextual fear memories, but in its absence (RE inactivation), there is reliance on an impoverished, non-hippocampal, fear memory system that imprecisely encodes context. Supporting this, Lin et al. (2020) demonstrated that the inactivation of RE/RH with muscimol significantly impaired the acquisition of trace fear conditioning in rats, which interestingly could be reinstated by *suppressing* RE prior to retrieval. Together these findings indicate that RE is directly involved in the acquisition of contexual fear memory as well as the “suppression” of a generalized (non-HF dependent) fear to aspects of the environment.

Moscarello (2020) described the interesting findings that (ventral) mPFC to RE projections suppress freezing behavior in a signaled active avoidance paradigm—as an adaptive response. Specifically, rats were trained to successfully avoid shock on an active avoidance task, which then reportedly reduces innate fear responses (e.g., freezing) to conditioned stimuli (CS) presented in a neutral setting. Moscarello (2020) reported that the inactivation of RE or chemogenetic suppression of mPFC-RE projections significantly increased freezing to CSs given in a neutral setting. This suggested that the mPFC-RE pathway may be responsible for inhibiting innate defensive behaviors (freezing) that would interfere with active coping responses in dangerous situations.

Maren et al. (Ramanathan et al., 2018b; Ramanathan and Maren, 2019) showed that inactivating RE with muscimol, or mPFC projections to RE using DREADDs, impaired fear extinction learning; that is, significantly increased freezing to conditioned tones during extinction training and during “retrieval” testing, 24 h after extinction learning. Further, RE cells were shown to fire at enhanced rates and levels of c-fos expression were greatly increased during extinction training. They proposed that extinction initiates an inhibitory process which prevents the retrieval of fear memory, and this is “mediated by projections from the mPFC to the hippocampus *via* the RE”.

Using various state of the art techniques in mice, Silva et al. (2021) recently reported that RE (or specially RE inputs to the amygdala) underlies the extinction of remote (30 day) but not recent (1 day) fear memories. Specifically, they showed: (1) that DREADD-induced activation or inhibition of RE, reduced or enhanced, remote fear memory, respectively; (2) that increases in RE activity were time-locked to the cessation of freezing; and (3) that optogenetic stimulation or inhibition of RE (or RE projections to the basolateral nucleus of the amygdala) decreased or increased freezing behavior in a remote fear extinction paradigm. Further, based on their demonstration that optogenetic activation of infralimbic (IL) cortical projections to RE also produced remote fear extinction, they proposed a IL → RE → BLA circuit for remote fear memory extinction.

Finally, Salay et al. (2018) described the involvement of RE (and RH), *via* projections to the amygdala and the prefrontal cortex, in innate fear showing that the activation of RE reduced fear behavior and increased tail-rattling (a sign of aggression) in mice. Complementing this, Linley et al. (2020) recently demonstrated that reversible suppression of RE increased anxiety-like behaviors on the elevated plus maze (EPM) which was accompanied by significantly increased levels of c-fos expression in RE (and RH). Taken together the foregoing findings indicate a critical role for RE in both learned and innate fear.

In summary, the inactivation of RE has been shown to disrupt WM/spatial working memory, executive processes and fear/avoidance behavior. While it is presently unknown whether the multiple, diverse functions of RE involve separate, or perhaps common (overlapping) regions of RE, it is clear that, as major interface between the HF and medial/orbital PFC, nucleus reuniens is intimately involved in the affective and cognitive functions served by these cortical structures.

## The dorsal midline thalamus: Paraventricular and paratenial nuclei

As stated, the dorsal midline thalamus consists of the PV and PT. PV is located medially below the third ventricle and dorsomedial to MD, and essentially spans the rostro-caudal extent of the thalamus. PT, however, is a rather small nucleus which lies lateral to PV at the rostral pole of the thalamus. Whereas, the projections of PV and PT significantly overlap (Vertes and Hoover, 2008), exceedingly few studies have examined the functional properties of PT independent of PV. As such, we focus on PV, describing its circuitry and its functional properties.

### Paraventricular nucleus of the dorsal midline thalamus: Circuitry

#### PV input

Similar to RE, PV receives a diverse array of afferents from the forebrain and the brainstem.

The main sources of subcortical input to PV are from structures of the brainstem and hypothalamus, with additional, but more limited, input from the amygdala, bed nucleus of stria terminalis (BST), the medial preoptic area (MPO) and the diagonal band nuclei (Sesack et al., 1989; Chen and Su, 1990; Hurley et al., 1991; Vertes, 1991, 1992, 2002; Otake and Nakamura, 1995; Otake et al., 1995; Vertes et al., 1995, 1999; Ruggiero et al., 1998; Novak et al., 2000; Krout et al., 2002; Goto and Swanson, 2004; Peng and Bentivoglio, 2004; Kirouac et al., 2005, 2006; Otake, 2005; Hoover and Vertes, 2011; Li and Kirouac, 2012).

While varying in density, brainstem afferents to PV derive from the ventral tegemental area (VTA), the pontomesencephalic RF, nucleus cuneiformis, the dorsal and median raphe nuclei, the PAG, the parabrachial complex (PB), the laterodorsal (LDT) and pedunculopontine (PPT) nuclei, the locus coeruleus (LC) and the solitary nucleus (NTS) (Chen and Su, 1990; Takada et al., 1990; Bester et al., 1999; Krout and Loewy, 2000a,b; Krout et al., 2002; Li and Kirouac, 2012). In a comprehensive examination of afferents to PV, Li and Kirouac (2012) observed considerably fewer brainstem inputs to PV than shown in previous reports (Krout et al., 2002), which they attributed to the relatively small size of their injections, confined to PV, and likely did not destroy fibers of passage. However, similar to previous studies, they identified labeled cells in the PB, PAG and the dorsal raphe nucleus, but surprisingly few in VTA, LC, and NTS.

Perhaps in contrast to the brainstem, PV receives input from several cell groups of the hypothalamus. They include the tuberomammillary, supramammillary, dorsomedial, posterior, lateral and parasubthalamic nuclei (Vertes, 1992; Vertes et al., 1995; Goto and Swanson, 2004; Kirouac et al., 2005, 2006; Li and Kirouac, 2012). The parasubthalamic nucleus, a preautonomic group implicated in visceral control, strongly distributes to PV, mainly to posterior PV (PVp) (Goto and Swanson, 2004). As well recognized, PV is also a major target of fibers from orexin (ORX) and cocaine and amphetamine-regulated transcript (CART)-containing cells of the lateral hypothalamus (Kirouac et al., 2005, 2006; Parsons et al., 2006; Matzeu and Martin-Fardon, 2018). While PV contains a rich plexus of dopaminergic (DA) fibers (Garcia-Cabezas et al., 2009), interestingly they reportedly originate from DA cell groups of the medial hypothalamus and the brainstem (DR/PAG) and not from major DA nuclei including VTA (Li et al., 2014). Finally, PV is essentially unique among midline nuclei in that it receives afferents from the suprachiasmatic nucleus (SCh) and the intergeniculate leaflet (Moore et al., 2000; Kawano et al., 2001) and as described below, PV sends return projections to SCh.

While the PV receives quite significant subcortical input from the brainstem and diencephalon, the major source of afferents to PV is from the cortex; prominently from the mPFC, agranular insular cortex and the ventral subiculum of the HF (Groenewegen, 1988; Sesack et al., 1989; Chen and Su, 1990; Hurley et al., 1991; Vertes, 2002, 2004; Jasmin et al., 2004; Hoover and Vertes, 2011; Li and Kirouac, 2012). Whereas, fibers throughout the mPFC project to PV, there is a dorsal to ventral gradient in density such that the anterior cingulate cortex (AC) distributes moderately, and PL and IL massively, to PV (Figures 2A–C). The ventral subiculum distributes quite selectively to the anterior PV, whereas the mPFC and insular cortex mainly target the posterior PV (Vertes, 2002, 2004; Li and Kirouac, 2012). Noting considerably stronger cortical than subcortical input to PV, Li and Kirouac (2012) suggested that this may be indicative a greater “top-down” influence on PV than generally recognized.

#### PV output

**Figure 6:**
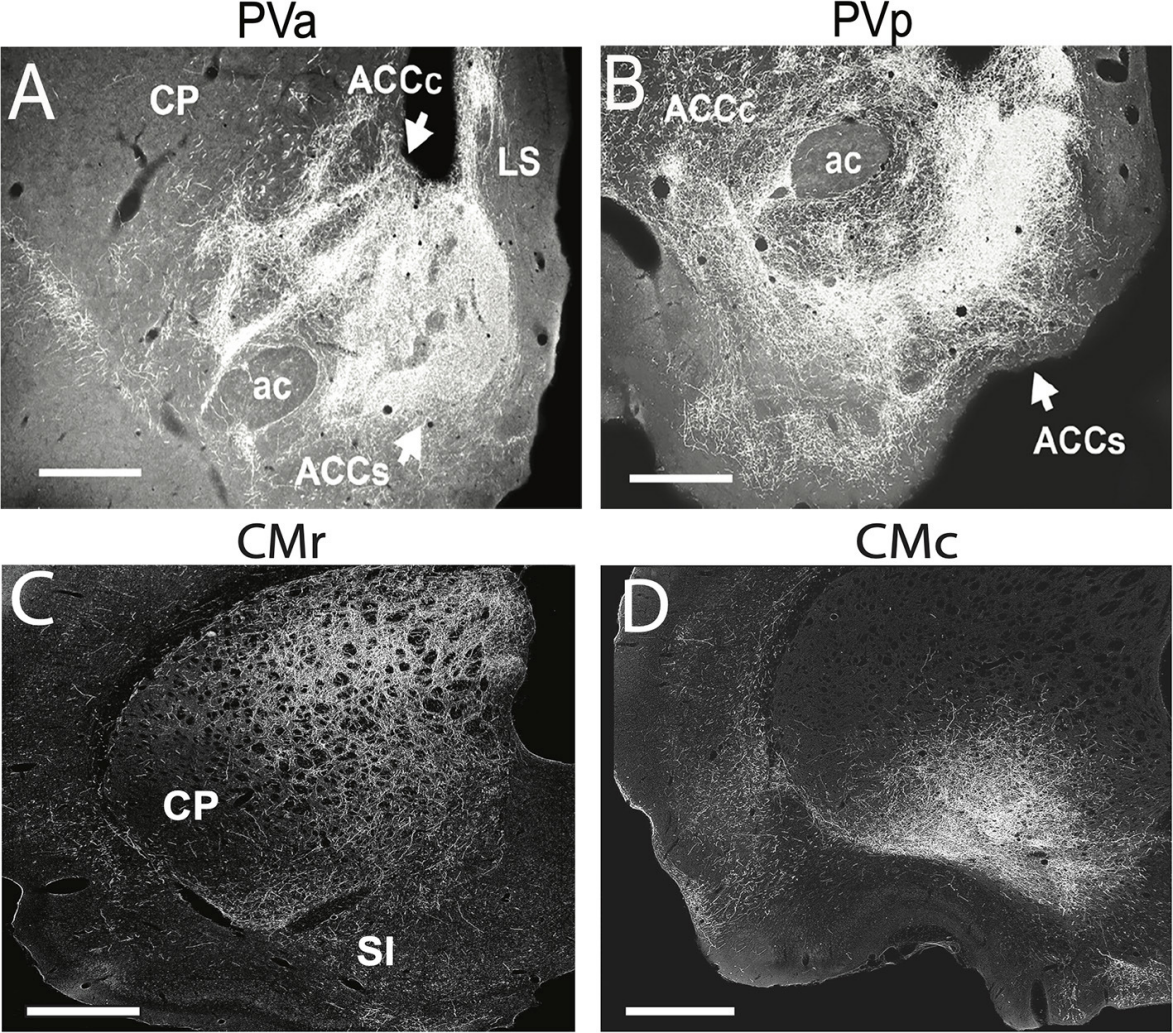
**(A,B)** Darkfield micrographs of transverse sections through the basal forebrain showing patterns of labeling in the nucleus accumbens (ACC) produced by anterograde tracer injections into the anterior (PVa) **(A)** and posterior (PVp) **(B)** paraventricular nucleus of thalamus of the rat. **(A)** Note the massive terminal labeling in the shell (ACCs) and core (ACCc) of ACC produced by a PVa injection. **(B)** Note the massive terminal labeling in the shell of ACC but less dense labeling in the core of AAC produced by the PVp injection. **(C,D)** Darkfield micrographs of transverse sections through the dorsal striatum (CP) depicting patterns of labeling produced by anterograde tracer injections in the rostral (CMr) **(C)** and caudal (CMc) **(D)** central medial nucleus (CM) of the thalamus of the rat. Note the pronounced terminal labeling in the dorsomedial quadrant of CP following the injection in CMr **(C)**, compared with the dense labeling confined to the ventrolateral sector of CP following the injection in CMc **(D)**. ac, anterior commissure; LS, lateral septum; SI, substantia innominata. Scale bar for **(A,B,D)** = 500 μm; for **(C)** = 750 μm. Modified from Vertes and Hoover (2008) and Vertes et al. (2012).

**Figure 7:**
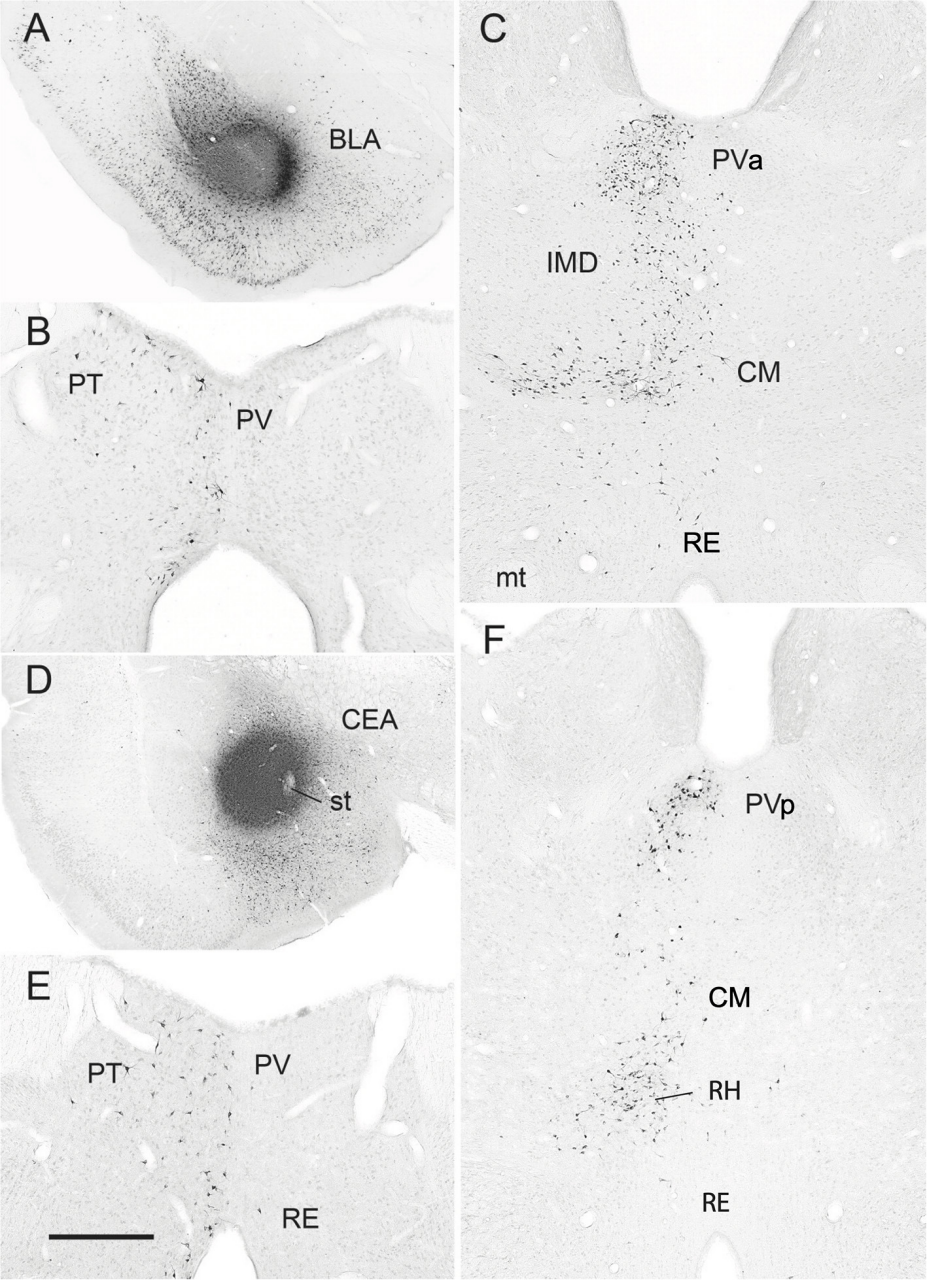
**(A–C)** Low-magnification bright-field micrographs of transverse sections through the forebrain depicting the site of a retrograde tracer (FluoroGold) injection in the basolateral nucleus (BLA) of the amygdala of the rat **(A)** and patterns of retrogradely labeled cells in the anterior paraventricular (PV) and paratenial (PT) nuclei of dorsal midline thalamus **(B)** and the posterior PV and central medial (CM) nuclei of the rostral intralaminar thalamus **(C)** produced by this injection. Note the significant numbers of retrogradely labeled neurons in the posterior PV and CM **(C)**, but fewer in the anterior PV, PT and nucleus reuniens (RE) **(B,C)** with this injection. **(D–F)** Low-magnification bright-field micrographs of transverse sections through the forebrain depicting the site of a retrograde tracer injection in the central nucleus (CEA) of the amygdala **(D)** and patterns of retrogradely labeled cells in the anterior PV, PT and RE nuclei of thalamus **(E)** and the posterior PV, CM and rhomboid (RH) nuclei of the thalamus **(F)** produced by this injection. Note moderate numbers of labeled cells in RE **(E)**, PT **(E)**, CM **(F)** and the anterior PV (**E**), but much denser clusters of cells in the posterior PV **(F)** and RH **(F)**. IMD, interomediodorsal nucleus of thalamus, mt, mammillothalamic tract; PVa, anterior paraventricular nucleus of thalamus; PVp posterior paraventricular nucleus of thalamus; st, stria terminalis. Scale bar for **(A)** = 750 μm; for **(B)** = 300 μm; for **(C)** =500 μm; for **(D)** =700 μm; for **(E)** = 400 μm; for **(F)** = 450 μm. Modified from Vertes and Hoover (2008).

PV distributes widely throughout the forebrain to cortical and subcortical structures (Berendse and Groenewegen, 1990, 1991; Meredith and Wouterlood, 1990; Su and Bentivoglio, 1990; Turner and Herkenham, 1991; Brog et al., 1993; Jasmin et al., 2004; Peng and Bentivoglio, 2004; Parsons et al., 2006, 2007; Hoover and Vertes, 2007; Li and Kirouac, 2008; Vertes and Hoover, 2008; Li et al., 2021b). The principal cortical targets of PV are the IL and PL cortices of the mPFC, the dorsal agranular insular cortex and the ventral subiculum of the hippocampus. The main subcortical PV projection sites are the claustrum, lateral septum, the core and shell of ACC (Figures 6A,B), olfactory tubercle, BST, the medial, basolateral (Figures 7A–C), central (Figures 7D–F) and cortical nuclei of the amygdala (AMY), and the suprachiasmatic (SCh), arcuate, and dorsomedial nuclei of the hypothalamus (Li and Kirouac, 2008; Vertes and Hoover, 2008). In addition, the caudal PV distributes modestly to the dorsal striatum (Vertes and Hoover, 2008; Hunnicutt et al., 2016).

Whereas, early reports described rather limited *collateral* PV projections to target structures (Bubser and Deutch, 1998; Otake and Nakamura, 1998), Kirouac et al. (Dong et al., 2017) demonstrated that PV distributes, *via* collaterals, to main terminal sites: the core and shell of ACC, BST and the basolateral and central nuclei of the AMY. Specifically, all combinations of dual retrograde injections yielded relative significant numbers of double labeled (collateralizing) cells in PV—about 7–17% per combinations of injection. Particularly striking was prominent PV projections to the shell of ACC; that is, ~80% of PV neurons were retrogradely labeled following dual injections in the dorsomedial and ventromedial shell of ACC and about 10% of them were double labeled—indicating collateral PV projections to both shell regions of the ACC. The anterior PV was shown to strongly target the dorsomedial shell of ACC and the posterior PV, the ventromedial shell of ACC. In like manner, tracing the axonal trajectory of individual PV neurons, Unzai et al. (2017) demonstrated that PV fibers ramify extensively throughout the ACC, while also branching to other sites, notably to the AMY and the mPFC. Finally, Viena et al. (2022) importantly described a small population of PV neurons with collateral projections to the subiculum of the HF and the mPFC.

### PV: Functional properties

#### PV: Functional properties—Overview

As PV represents a critical interface brainstem/diencephalic and forebrain limbic structures (Kirouac, 2015), PV has been linked to various functions, including arousal, feeding/appetitive behavior, fear/aversion and drug addiction (for review, Hsu et al., 2014; Kirouac, 2015; Millan et al., 2017; Huang et al., 2018; Zhou and Zhu, 2019; Barson et al., 2020; McGinty and Otis, 2020; Iglesias and Flagel, 2021; McNally, 2021; Penzo and Gao, 2021; Bu et al., 2022). These behaviors may have a common thread suggesting a key role for PV in the appetitive and aversive motivated behaviors.

An early groundbreaking report by Kelley et al. (2005) identified PV as an integral part of a hypothalamic-thalamo-striatal circuit subserving appetitive/reward behavior. According to their model, PV receives information from the hypothalamus related to reward, energy demands, circadian rhythms, and behavioral states and relays it mainly to the ACC to initiate/drive motivated behaviors, prominently hedonic feeding. With revisions, this model has served as a framework for considerable subsequent research on PV functions. We will focus on PV's role in feeding, drug addiction and arousal.

#### PV functional properties—Feeding/motivated behavior

The role of the PV in feeding behavior is complex and appears to involve separate afferents to the anterior (PVa) and posterior PV (PVp), with correspondingly differential effects on the ACC in the control of feeding. Specifically, it has been shown that the activation, or alternatively the suppression of PV can promote feeding, likely *via* different circuitries (for review, Petrovich, 2021).

Early evidence favoring the activation of PV in feeding stemmed, at least in part, from the demonstration that PV is a major recipient of orexin fibers (Kirouac et al., 2005) and ORX serves a well-recognized role in feeding behavior (for review, Barson and Leibowitz, 2017). For instance, Choi et al. (2010) initially demonstrated that ORX-receptor containing cells of PV were activated by the anticipation of food rewards, and subsequently (Choi et al., 2012) that injections of ORX-A into PV increased dopamine levels in the ACC, while reductions of ORX-1 receptor signaling in PV suppressed hedonic feeding in rats. They concluded that PV is critical for mediating the actions of orexin “on brain dopamine and reward based feeding.” More recently, Barson et al. (2015) described the interesting findings that ORX exerted differential effects on the PVa and the PVp; that is, injections of ORX-A in the PVa elicited ethanol drinking, whereas injections in the PVp enhanced the intake of sucrose.

Consistent with the foregoing, Meffre et al. (2019) identified the posterior PV as a critical node in relaying “hunger-related” signals from hypothalamic ORX cells to the ACC in feeding behavior. They demonstrated that: (1) satiety reduced the activity of PVp (and ACC) neurons to cues signaling rewards; (2) blockade of ORX-2 receptors in PVp suppressed responses to food rewards in hungry rats; and (3) injections of ORX-A or optogenetic PV stimulation restored feeding in sated rats (Meffre et al., 2019). In effect, information on metabolic need from ORX neurons was conveyed, *via* PV, to the ACC to initiate feeding in the presence of palatable food. Supporting these findings, Sofia Beas et al. (2020) reported that optogenetic activation of catecholaminergic-containing fibers of the ventrolateral medulla projecting specifically to PVp elicited feeding in sated mice, while optogenetic silencing of these fibers attenuated feeding.

Associated with, and possibly complementing ORX, agouti-related protein (AGRP) appears to exert a critical influence on PV in the regulation of feeding behavior (Gropp et al., 2005; Wu et al., 2009b; Betley et al., 2013). For instance, Betley et al. (2013) reported that optogenetic stimulation of AGRP+ fibers of the arcuate nucleus that project to PV significantly increased food consumption—and over several sessions. More recently, Wang et al. (2021) showed that the ablation or optogenetic inhibition of AGRP+ fibers projecting to PV suppressed food seeking behavior in food restricted but not in sated mice.

Seemingly in direct contrast to the foregoing findings, several reports have shown that the “suppression” (or inactivation) of PV stimulates feeding behavior (Bhatnagar and Dallman, 1999; Stratford and Wirtshafter, 2013; Zhang and van den Pol, 2017; Reed et al., 2018; Otis et al., 2019). For example, Bhatnagar and Dallman (1999) initially demonstrated that PV lesions produced significant increases in food intake and weight gain, while Stratford and Wirtshafter (2013) showed that injections of muscimol into PV, dose-dependently, increased the intake of food in non-deprived rats.

In a multifaceted study using cre-dependent mice, Zhang and van den Pol (2017) reported that the suppression of PV, produced by inhibitory inputs from the zona incerta (ZI) to PV, gave rise to robust increases in food intake and weight gain—described as “binge-like” eating. Specifically, activation of GABAergic ZI cells or GABAergic terminals in PV produced an immediate and sustained increase in feeding behavior, while the selective ablation of GABAergic ZI cells, or glutamatergic PV neurons, produced long term increases in food intake—for up to 16 weeks with the PV lesions. Finally, stimulation of excitatory inputs to PV or chemogenetic activation of PV glutamatergic neurons reduced food intake.

Using calcium imaging based fiber photometry, Reed et al. (2018) examined the effects on food consumption of excitatory inputs to the shell of ACC from the dorsal midline thalamus, the basolateral nucleus (BLA) of AMY, and the ventral HF in mice, and showed that reductions in activity from each of these sites to the (rostral) ACC generated feeding behavior—with largest reductions in activity (per site) seen with visits to food ports. In addition, optogenetic suppression of each input to the ACC significantly enhanced food intake. It was concluded that reductions of excitatory activity to the rostral ACC from these three sources, individually or combined, is responsible for driving feeding behavior.

Using similar imaging techniques combined with a classical conditioning paradigm in mice, Otis et al. (2019) described marked decreases in PV activity to cues signaling rewards (sucrose), which interestingly led to a strengthening of cue (CS+)-reward associations. Specifically, reductions in PV activity to CSs for reward resulted from: (1) a CS-elicited inhibition of glutamatergic PFC cells projecting to PV; and (2) the activation of GABAergic cells of the lateral hypothalamus (LHy) innervating PV. Further, decreases in prefrontal-PV activity were linked to the presentation of cues (CSs), whereas increases in (GABAergic) LHy-PV activity were tied to licking behavior. It was concluded that the PV integrates information from the PFC (cue-reward associations) with that from the LHy (response to reward), to initiate feeding, *via* actions on the ACC.

In summary, the activation or the suppression of PV can induce feeding behavior—which appears driven by separate hypothalamic systems: an excitatory peptidergic (ORX and CART) system for activation-induced feeding, and an inhibitory ZI and LHy system that suppresses feeding. As PVa and PVp projections differ (see above), this dichotomy in PV's role in feeding could be attributed to the differential involvement of the PVa and PVp in feeding behavior. This was, in fact, proposed by Meffre et al. (2019) stating that there is “growing evidence indicating opposite effects of these two [PV] subterritories on reward seeking”. Although oversimplified, the evidence reviewed above suggests that PVp mainly monitors the metabolic state (hunger) of the animal, whereas the PVa primarily serves to initiate behavioral responses to cues signaling food reward.

#### PV functional properties—Drug seeking and reinstatement

There is a clear overlap in systems controlling feeding and drug abuse, including PV, which supports the view that addictive drugs act through the natural reward circuitry. A leading advocate of this position, Martin-Fardon and Boutrel (2012) stated: “the neural circuitry encoded for natural rewards is usurped by drugs of abuse. Neuroplasticity within this neural circuitry is believed to be responsible for the maladaptive (compulsive) behavior characteristic of addiction.”

Attention has only recently focused on the role of PV in drug abuse. In early reports, Deutch and colleagues (Deutch et al., 1998; Young and Deutch, 1998) described enhanced levels of c-fos expression in PV to the delivery of amphetamine or cocaine, and further that PV lesions blocked cocaine-induced locomotor sensitization. Hamlin et al. (2009) similarly reported marked increases in numbers of c-fos+ cells in PV with the reinstatement of alcohol seeking behavior, and additionally that PV lesions prevented this reinstatement. In like manner, Dayas et al. (James et al., 2010, 2011; Yeoh et al., 2014) demonstrated that the inactivation of PV with tetrodotoxin (TTX) or CART suppressed the reinstatement of cocaine seeking behavior in rats (James et al., 2010), and that the degree of reinstatement to cocaine was correlated with levels of c-fos expression in PV (James et al., 2011). Finally, in the slice preparation in mice, Yeoh et al. (2014) reported that cocaine pretreatment, compared to controls, enhanced the excitability of PV neurons which was suppressed by CART peptides.

Consistent with the foregoing, injections of GABA agonists into PV, but not into adjacent regions of the thalamus, were shown to block the expression of cocaine-induced place preference in rats (Browning et al., 2014). Neumann et al. (2016) demonstrated that selective disruption of PV-ACC projections significantly decreased cocaine self-administration in rats, and importantly this was accompanied by increases in silent synapses in ACC which returned to baseline following a prolonged period of withdrawal. The authors concluded that the PV-ACC projection was “essential for acquisition of cocaine self-administration” (Neumann et al., 2016).

In addition to orexin's well-established involvement in feeding behavior (see above), ORX serves an equally important role in drug-related behaviors—including ORX actions on PV (Harris et al., 2005; Jupp et al., 2011; Martin-Fardon and Boutrel, 2012; Matzeu et al., 2014; Matzeu and Martin-Fardon, 2022). For instance, early reports described increases in c-fos expression of orexin-PV projecting cells following the exposure to nicotine (Pasumarthi and Fadel, 2008) or to cues signaling alcohol (Dayas et al., 2008).

In a series of studies, Martin-Fardon and colleagues examined ORX actions on the PV in cocaine seeking behavior, comparing effects to natural rewards (Matzeu et al., 2015, 2016; Martin-Fardon et al., 2016). In an initial study, rats were trained to associate cues (CSs) with cocaine or a natural reward (sweetened condensed milk) (SCM), and after a period of extinction, the CSs were reintroduced, and it was reported that infusions of GABA agonists into the PVp prevented the reinstatement of cocaine seeking behavior but had no effect on SCM seeking (Matzeu et al., 2015). In a follow-up examination of the effects of orexin on cocaine reinstatement in rats, Matzeu et al. (2016). showed that: (1) injections of ORX-A into the PVp reinstated primed cocaine seeking behavior; and (2) the co-administration of ORX-A with ORX-1 receptor antagonists did not prevent reinstatement, whereas co-injections with ORX-2 receptor antagonists blocked cocaine seeking behavior—indicating a (selective) involvement of ORX-2 receptors in cocaine-mediated actions on PV. More recently, Matzeu and Martin-Fardon (2020) demonstrated that blocking the effects of ORX on the PVp prevented the reinstatement of ethanol and SCM seeking behaviors in alcohol-addicted rats, indicating that ORX in PV may serve a role in the reinstatement to both drugs of abuse and natural rewards.

Examining the role of PV in opiate addiction, Keyes et al. (2020) described two distinct outputs from PV contributing to morphine-induced conditioned place preference (CPP) in mice: a PV to central nucleus (CeA) of AMY pathway, and PV to ACC projection for the acquisition and persistence of CPP, respectively. Specifically, chemogenetic suppression of the PV to ACC pathway prevented the reinstatement of place preference to morphine—an effect that lasted for 24 h. The authors concluded that morphine-induced modifications of the PV-ACC circuitry serves to “maintain the contextual association and drive morphine seeking.” In effect, this system appears to contribute to relapse to morphine and its suppression may prevent relapse. In summary, the PV appears to serve a critical role in the acquisition, maintenance, extinction, and reinstatement of drugs of abuse—mainly through actions on the ACC.

#### PV functional properties—Arousal

In addition to effects on feeding and drug seeking behavior, ORX also serves a well-established role in arousal/wakefulness. For instance: (1) ORX cells of the LHy fire at high rates during active wakefulness, and at significantly reduced rates during drowsy or sleep states; (2) ORX agents/agonists produce prolonged periods of wakefulness, while ORX antagonists significantly increased amounts of NREM and REM sleep; (3) ORX mutant (KO) mice cannot maintain long periods of wakefulness and repeatedly vacillate between sleep/wake states; (4) ORX cells are reciprocally connected to all “arousal-related” nuclei of the brain; and (5) deficits in ORX signaling produces narcolepsy in rodents, dogs and humans (for review, Peyron et al., 2000; Sakurai et al., 2010; Alexandre et al., 2013; Li et al., 2018).

While ORX exerts actions at multiple sites of the brain in arousal, recent reports have identified PV as an important target contributing to arousal/wakefulness. For instance, in an early report, Peng et al. (1995) observed significantly greater numbers of c-fos labeled cells in PV in waking than in sleep in rats. Ren et al. (2018) similarly reported enhanced levels of c-fos expression in PV during waking in mice, and further demonstrated in behaving mice that PV cells fire at much greater rates in waking (7–10 Hz) than in NREM sleep (1–4 Hz), with characteristic increases or decreases preceding sleep-wake or wake-sleep transitions, respectively. They further showed, using a combination of techniques, that chemogenetic suppression or lesions of PV produced significant reductions in wakefulness during the dark (active) phase of mice, while optogenetic PV stimulation during the light phase produced rapid transitions from NREM or REM sleep to wakefulness. The PV was described as integral part of an (excitatory) circuit for wakefulness—with PV driven by ORX input from the hypothalamus and, in turn, exerting excitatory actions on the ACC in waking. Finally, noting that the PV has been linked to various behaviors including feeding, drug addiction and fear conditioning, Ren et al. (2018) remarked that each of these behaviors “require elevated wakefulness”.

In accord with the foregoing, Matyas et al. (2018) described the involvement of calretinin-containing (CR) cells of the dorsomedial thalamus (DMT), mainly PV, in arousal. In a multipart report, they demonstrated that DMT-CR+ cells discharge at elevated rates immediately before the transition from sleep to wakefulness, and that optogenetic DMT stimulation produced rapid awakenings from NREM or REM sleep. Interestingly, they further showed that short duration (1 s) DMT stimulation during sleep mimicked the natural-occurring micro-arousals of that state, whereas longer duration stimulation (10 s) produced extended periods of wakefulness, accompanied by active locomotion. Finally, they demonstrated that DMT-CR neurons distribute, *via* collaterals, to several forebrains sites, and that these branching DMT cells simultaneously activate various forebrain regions – an effect which is “optimal to elicit a generalized brain wide effect like arousal.” They concluded that DMT-elicited arousal “is a necessary component of the active execution of any given behavior” (Matyas et al., 2018). Following up on this view, Otis et al. (2018) speculated that PV's involvement in feeding may be attributed to a heightened state of arousal rather feeding per se, stating that the PV circuitry “may contribute to reward processing by inducing a state of arousal or wakefulness rather than specifically driving reward seeking or consumption”. In this regard, Yamanaka et al. (2003) proposed that orexin cells register metabolic needs and under conditions of deprivation (e.g., fasting) ORX cells drive adaptive responses to satisfy those needs—or fasting induces arousal which triggers food seeking behavior. Supporting this, they showed that ORX activity was suppressed by signals for satiety and activated by those for hunger, and mutant mice, with ablated ORX cells, failed to show typical increases in arousal/wakefulness and locomotor activity to fasting. Coupling CR activity (or calretinin+ PV neurons) with hunger-induced arousal, Hua et al. (2018) showed that 24 h of fasting or injections of ghrelin, a hormone signaling hunger, profoundly elevated c-fos levels in PV neurons, mainly in CR+ cells. They further showed that optogenetic stimulation of CR+ PV cells projecting to BST in CR-Cre mice significantly increased wakefulness. Together this demonstrates a significant excitatory role for CR+ PV neurons in signaling hunger as well as arousal.

Gao et al. (2020) recently identified two genetically and anatomically distinct subtypes of PV cells. They showed that cells expressing the dopamine D2 receptor (Type I cells) were mainly concentrated in the posterior PV, whereas cells lacking this receptor (Type II cells) were most numerous in the anterior PV. They further importantly showed: (1) that Type I cells are reciprocally connected with PL and Type II cells with IL of the mPFC, and (2) that stimulating Type II cells (non-DA) decreases arousal – suggesting that suppressing them may promote arousal.

Finally, Martin-Fardon and colleagues (Matzeu et al., 2016) speculated that ORX effects on PV in the reinstatement of cocaine-seeking behavior could involve to ORX's actions on arousal. They noted that ORX actions on PV induce “cortical activation that is linked to general arousal, which could explain the reinstatement of cocaine-seeking.” In effect, appropriate levels of arousal may be a necessary backdrop for feeding and drug seeking behavior.

## Comparisons of anatomical and functional properties of the dorsal (PV) and ventral (RE) midline thalamus

Whereas, the midline (and ILt) nuclei of the thalamus were initially thought to project widely throughout the cortex and exert rather undifferentiated effects on behavior, it has recently been shown that each of the midline/ILt nuclei exhibit a unique pattern of projections and participate in distinct functions. In this regard, the differences between RE and PV projections and functions are striking. While RE and PV share common inputs, projections to RE are more widespread and diverse, especially from the brainstem and hypothalamus. With respect to output, there are major differences in RE and PV projections. Specifically, RE almost solely targets limbic cortical structures, such as the orbitomedial, insular, retrosplenial, and parahippocampal cortices and the HF, and minimally subcortical sites, mainly projecting to the rostral ACC. By contrast, PV predominantly distributes to limbic subcortical sites, including the septum, BST, olfactory tubercle, ACC, amygdala, and hypothalamus—with cortical projections essentially limited to the ventral mPFC (IL and PL) and ventral subiculum.

The functional properties of RE and PV parallel their respective projections to limbic cortical and subcortical sites; that is, RE is primarily involved in cognitive functions and PV in motivated behaviors. As reviewed, RE has been shown to serve a critical role in various cognitive functions to include working memory/SWM, executive functions (attention, behavioral flexibility, reversal learning, decision making) and contexual fear memory. The role of RE in cognitive functions appears largely dependent on RE's position as a key interface between the HF and the mPFC (and ORB)—in the two-way exchange of information between these structures. As described, RE-mediated disruptions of communication between the hippocampus and the mPFC or ORB produces deficits in SWM, executive functions, and contextual fear memory.

By contrast, PV has been shown to serve a crucial role in motivated behaviors. We focused on PV's involvement in appetitive functions: feeding, drug addiction and arousal. The PV has been shown to be an integral part of hypothalamic-thalamo-ventral striatal circuit subserving appetitive behaviors. With respect to feeding, both the activation and suppression of PV induces feeding, putatively through discrete actions on PVp and PVa, respectively, controlling different aspects of feeding. With respect to drug addiction and arousal, ORX input from the hypothalamus to PV has been shown to exert a potent influence on PV in these behaviors. Various manipulations that alter ORX actions on the PV suppress drug seeking behavior and reinstatement, and dampen arousal responses in PV.

While the dorsal (PV) and ventral (RE) midline thalamus largely serve separate roles in cognitive and motivational behaviors, PV and RE also commonly participate in certain functions, notably, in affect/fear and arousal. For instance, RE is recruited in unlearned fear and anxiety, in addition to conditioned learned fear, whereas PV, as reviewed, participates in various appetitive/aversive behaviors but also has recently been linked to innate and learned fears, as well as anxiety (Li and Kirouac, 2008; Li et al., 2010; Kirouac, 2015, 2021; Penzo et al., 2015; Do Monte et al., 2016; Barson et al., 2020; Penzo and Gao, 2021). Regarding arousal, both PV and RE receive afferents from the brainstem involved in arousal and sleep-wake control. While brainstem inputs to PV complement ORX projections to PV in arousal, convergent inputs to RE from the brainstem, hypothalamus and limbic forebrain underscore RE's involvement in vigilance, attention and sleep/wake states (Viena et al., 2021).

## Rostral intralaminar nuclei (central medial, paracentral, central lateral)

The intralaminar nuclei (ILt) of thalamus encompass a collection of nuclei located in the medial and dorsal part of the thalamic complex. The intralaminar thalamic nuclei are located lateral to the mediodorsal nucleus and “embedded” within the internal medullary lamina. As previously indicated, the intralaminar nuclei are divided into a rostral and caudal division, with the rostral group consisting of the central medial (CM), paracentral (PC), and central lateral (CL) nuclei. We discuss the circuitry and function of the rostral intralaminar nuclei, with an emphasis on CM, as its connections more closely parallel those of the midline thalamic nuclei than do other ILt nuclei.

### Rostral intralaminar nuclei: Circuitry

#### ILt input

The main sources of afferents to the rostral ILt arise from structures/regions of the brainstem and cortex. The following brainstem nuclei project to the rostral ILt: dorsal and median raphe nuclei (Vertes, 1991; Hermann et al., 1996; Morin and Meyer-Bernstein, 1999; Vertes et al., 1999, 2010; Krout et al., 2002; Muzerelle et al., 2016; Urban et al., 2016), locus coeruleus (Jones and Yang, 1985; Krout et al., 2002), pedunculopontine (PPT) and laterodorsal tegmental (LDT) nuclei (Hallanger et al., 1987; Hallanger and Wainer, 1988; Bolton et al., 1993), the ventral tegmental area (Beckstead et al., 1979; Krout et al., 2002), parabrachial complex (Bester et al., 1999; Krout and Loewy, 2000a; Bourgeais et al., 2001; Iwai et al., 2015; Deng et al., 2020), periaqueductal gray (Cameron et al., 1995; Krout and Loewy, 2000b; Kincheski et al., 2012; Sun et al., 2020), superior colliculus (Yamasaki et al., 1986; Krout et al., 2001), nucleus incertus (Goto et al., 2001; Olucha-Bordonau et al., 2003), the dorsal horn of the spinal cord (Li et al., 2021a) and dense projections from the mesencephalic, pontine, and medullary reticular formation (Glenn and Steriade, 1982; Vertes et al., 1986; Vertes and Martin, 1988; Villanueva et al., 1998; Krout et al., 2002). The rostral ILt nuclei also receive significant, but more limited, input from diencephalic structures including the reticular nucleus of thalamus (Velayos et al., 1989; Kolmac and Mitrofanis, 1997), the zona incerta (Power et al., 1999; Power and Mitrofanis, 2001), the substantia nigra pars reticulata (McElvain et al., 2021), and the lateral and supramammillary nuclei of the hypothalamus (Vertes, 1992; Peyron et al., 1998).

Regarding cortical afferents, the PFC is a prominent source of projections to the rostral intralaminar nuclei, with differences in afferents to CM, CL, and PC (Reep et al., 1987; Sesack et al., 1989; Reep and Corwin, 1999; Vertes, 2002, 2004; Jasmin et al., 2004; Hoover and Vertes, 2011; Prasad et al., 2020). For instance, the mPFC (IL, PL and AC) distributes moderately to CM but minimally PC and CL (Figures 2A–C), whereas the dorsally located AGm prominently targets PC and CL but avoids CM (Vertes, 2002, 2004). By comparison, the orbital and insular cortices distribute moderately to the rostral ILt, with heaviest projections from the medial orbital cortex (MO) to CM (Figure 2D) (Shi and Cassell, 1998; Jasmin et al., 2004; Hoover and Vertes, 2011). With respect to sensorimotor cortical afferents to ILt, Prasad et al. (2020) recently showed for mice that the motor cortex (M1) distributes to the entire intralaminar complex, whereas somatosensory (SI) and visual (V1) cortices essentially only project to CL.

#### ILt output

The principal targets of the intralaminar nuclei are the cortex and the striatum. In general, CL and PC mainly innervate sensorimotor regions of the cortex and the dorsal striatum, whereas CM distributes over a much wider region of the forebrain to both limbic and non-limbic sites (Berendse and Groenewegen, 1990, 1991; Conde et al., 1990, 1995; Su and Bentivoglio, 1990; Hicks and Huerta, 1991; Turner and Herkenham, 1991; Brog et al., 1993; Reep and Corwin, 1999; Erro et al., 2002; Van der Werf et al., 2002; Jasmin et al., 2004; Wang and Shyu, 2004; Hoover and Vertes, 2007; Vertes et al., 2012).

With some overlap, there is medial to lateral gradient in PC and CL projections to the dorsal PFC such that PC mainly targets the anterior cingulate (AC) cortex and CL the adjacent secondary motor cortex (AGm). More specifically, PC primarily distributes to the dorsal and ventral AC, and secondarily to AGm and caudally to the retrosplenial (RS) cortex—with minimal projections to other cortical regions. CL mainly targets AGm, with additional projections to the primary motor cortex (AGl), primary and secondary somatosensory cortices, the retrosplenial cortex, and the occipital cortex (Berendse and Groenewegen, 1991; Conde et al., 1995; Reep and Corwin, 1999; Wang and Shyu, 2004; Hoover and Vertes, 2007; Ahrlund-Richter et al., 2019). However, slightly at odds with previous reports, Xue et al. (2022), using viral tracing techniques in mice, identified stronger inputs to AC from CL than from PC or CM.

Similar to the output to the cortex, intralaminar fibers project to separate but overlapping regions of the dorsal striatum (caudate-putamen, C-P). PC and CL distribute to the dorsomedial and dorsolateral striatum, respectively, and hence as a pair, encompass the entire dorsal half of the striatum. The PC and CL innervate medium spiny neurons (MSNs) of the dorsal striatum (Castle et al., 2005; Doig et al., 2010; Ellender et al., 2013) as well as cholinergic and GABAergic interneurons (Smith et al., 2004; Ding et al., 2010; Arias-Garcia et al., 2017; Klug et al., 2018). Thalamocortical and thalamostriatal connections are also highly topographically organized such that the projections of individual intralaminar nuclei reach specific regions of the cortex and the striatum which are also interconnected *via* corticostriatal projections (Groenewegen et al., 1999; Groenewegen and Witter, 2004). For instance, CL selectively targets the medial agranular cortex (AGm) and the dorsolateral quadrant of C-P, and AGm, in turn, distributes dorsolaterally to the C-P (Berendse and Groenewegen, 1990, 1991; Wu et al., 2009a). As a result, CL is positioned to directly affect AGm as well as its target zone in the striatum. These highly organized cortico-striatal-thalamic networks show a high degree of convergence. For instance, Deschênes et al. (1996) traced single intralaminar fibers and showed that ILt cells send collateral projections to the striatum and cortex, while Huerta-Ocampo et al. (2014) demonstrated that both cortical and thalamic axons converge onto medium spiny striatal neurons. Regarding the latter, Huerta-Ocampo et al. (2014) proposed that “the ensemble of MSNs that fire during a basal ganglia-associated behavior is a consequence of activity in corticostriatal neurons carrying motor and cognitive information and activity in thalamostriatal afferents carrying information on saliency and wakefulness”.

Finally, though not as pronounced as the dorsal striatum, the ventral striatum receives input from the ILt, which preferentially targets the lateral core of ACC, with most pronounced projections arising from PC (Berendse and Groenewegen, 1990, 1991; Brog et al., 1993; Erro et al., 2002; Li et al., 2018). It appears that the intralaminar thalamus can also indirectly influence the ventral striatum *via* the mPFC. Specifically, Cruz et al. (2021) recently mapped prefrontal (PFC) projections to the ACC and identified significant numbers of ILt cells that project to PL neurons distributing to ACC.

Similar to the other intralaminar nuclei, the main output of CM is to the cortex and dorsal and ventral striatum, but.CM also targets a diverse set of limbic forebrain structures, prominently the amygdala (Van der Werf et al., 2002; Hoover and Vertes, 2007; Vertes et al., 2012; Amir et al., 2019). Vertes et al. (2012) examined rostral and caudal CM projections and noted marked differences in their patterns of distribution. The rostral CM projects to the following structures: the orbitofrontal PFC, including AGm, AC, prelimbic, dorsolateral orbital, and dorsal agranular insular cortices; the entire dorsal striatum (Figure 6C), the shell and core of ACC and the basolateral nucleus (BLA) of AMY (Figures 7A,C).

**Figure 8:**
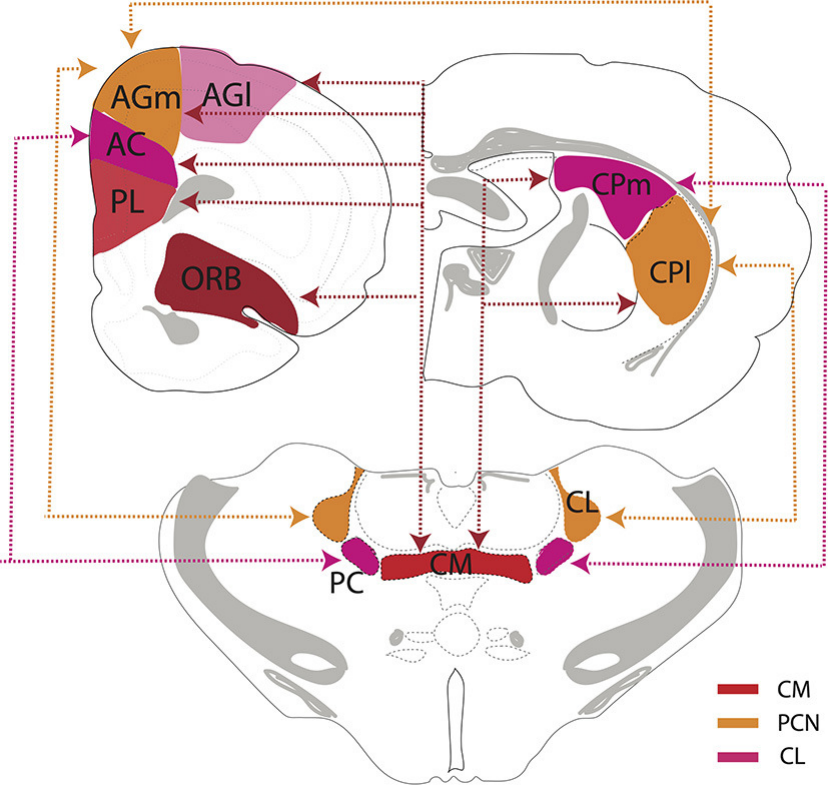
Schematic representation depicting the interconnections/circuitry between the rostral intralaminar thalamus, the dorsal striatum, the medial prefrontal, orbital and frontal motor cortices. The paracentral (PC) and central lateral (CL) nuclei are reciprocally linked to separate but overlapping regions of the frontal cortex and dorsal striatum. CL is reciprocally linked to the secondary motor cortex (AGm) and the lateral aspect of dorsal striatum (CPl) and AGm and CPl in turn reciprocally connected. By comparison, PC is reciprocally linked to the anterior cingulate cortex (AC) and medial aspect of the dorsal striatum (CPm), and AC and CPm are, in turn, reciprocally connected. By contrast with CL and PC, the central medial nucleus (CM) is much more widely interconnected with striatal-cortical circuitry as CM is reciprocally connected with entire frontal/prefrontal cortex (PL, AC, AGm, AGl), the medial and lateral dorsal striatum and add additionally the orbital cortex (ORB). Accordingly, CM may represent a conduit linking striatal, limbic and motor systems of the forebrain. PL, prelimbic cortex, AGl, primary motor cortex.

By comparison, the caudal CM mainly targets the lateral and dorsolateral orbital cortices, the dorsal and ventral agranular insular cortices, the gustatory/visceral cortex, primary somatosensory and motor cortices, and the perirhinal cortex. Further, the caudal CM primarily distributes to lateral/ventrolateral regions of C-P (Figure 6D) and lacks projections to ACC. Finally, unlike the rostral CM which mainly targets BLA, the caudal CM distributes more widely throughout the AMY to anterior, lateral, central, medial, cortical, and basal divisions (Figures 7D,F) (Vertes et al., 2012). Figure 8 summarizes the highly topographically organized corticostriatal circuitry of the rostral ILt with the mPFC (PL, AC), the primary and secondary motor cortices (AGl, AGm), the orbital cortex and medial and lateral of the dorsal striatum.

In summary, while CM, CL, and PC of the rostral ILt project to distinct subregions of the cortex, striatum and amygdala, together they distribute widely over these regions, to virtually blanket the cortical mantle, entire C-P, the ventral striatum and the amygdala.

### ILt: Functional properties

#### ILt functional properties—Overview

Owing to the complex configuration of ILt nuclei (Figure 1), their intricate relationship to each other (and to neighboring thalamic nuclei) and their shared circuitry, it has been difficult to separately investigate the functions of each of the ILt nuclei. Moreover, most analyses of these nuclei have described overlapping functions. As such, we discuss the rostral ILt collectively, noting differences when available. The ILt, through extensive projections to the striatum and cortex participates in a range of behaviors including sensorimotor coordination, pain modulation, arousal and cognition. Presently, we will address the role of ILt in limbic associated functions: arousal/wakefulness and cognition.

#### ILt functional properties—Arousal and consciousness

As described, ILt nuclei receive a vast and diverse array of input from the brainstem, particularly from the brainstem RF, and, in turn, are the source of projections to the prefrontal, sensory, and motor cortices. This initially led to the view, subsequently supported, that ILt bridges the effects of the brainstem on the cortex – or is an integral part of the ascending reticular activating system (ARAS), responsible for states of arousal/consciousness (Moruzzi and Magoun, 1949; Jones, 2003; Yeo et al., 2013; Gao et al., 2019). In an early study, Glenn and Steriade (1982) reported that CL neurons in cats, which were activated by midbrain RF stimulation and antidromically driven from the cortex, fired at high tonic rates of activity in waking (W) and REM sleep and at low rates in slow wave sleep (SWS). Accordingly, they concluded that CL cells serve “a role in the tonic activation processes” producing cortical arousal. More recently, Gent et al. (2018) similarly found that CM cells, like those of CL, discharged at significantly higher rates in waking and REM sleep than in SWS—with the highest rates in REM sleep. In addition, optogenetic stimulation of CM, but not that of the ventrobasal complex (VB), aroused sleeping mice, producing a rapid transition to wakefulness (Gent et al., 2018).

Schiff et al. in a series of studies directly linked the central thalamus (mainly CL) to processes of arousal and consciousness (for review, Shah and Schiff, 2010). For instance, Shirvalkar et al. (2006) reported that CL stimulation in rats produced widespread cortical activation and enhanced performance on an object recognition task. Liu et al. (2015) subsequently showed that high frequency stimulation of CL activated the cortex and aroused sleeping rats, whereas low frequency stimulation suppressed cortical activity and promoted sleep. In humans, Schiff et al. (2007) described the remarkable findings that deep brain stimulation (DBS) of the central thalamus restored consciousness and cognitive processing of a patient in a minimally conscious state (MCS). Since this hallmark paper, several subsequent reports have confirmed that DBS of the intralaminar complex improves processes of consciousness and awareness in MCS and vegetative-state patients (Schiff et al., 2007, 2009; Chudy et al., 2018, 2020). The arousal and behavioral enhancement of this clinical constellation appears to be linked to CL, and not to other regions of ILt, as these effects were recently mimicked by CL stimulation in healthy non-human primates (Janson et al., 2021).

This restorative effect and cortical enhancement of ILt/central thalamus has also been highlighted using rodent models. Lin et al. (2016) demonstrated that CL stimulation in rats increased c-fos expression across motor, anterior cingulate and parietal cortices, the dorsal and ventral striatum, and the hippocampus. Additionally, CL stimulation synchronized theta/alpha oscillations between the thalamus and striatum (thus strengthening their connections), upregulated dopamine D2 and cholinergic receptors in C-P and improved performance on an instrumental conditioning task.

Finally, the rostral ILt, centered in CM, appears to be important site for the actions of general anesthetics (GAs). Baker et al. (2014) described marked reductions in the discharge frequency of CM neurons in rats in the transition from waking to non-REM sleep (NREM), and also following the loss of consciousness to the administration of general anesthetics (GAs). Additionally, noradrenergic input from the LC to CM appears to be a major excitatory drive to CM in arousal, as its suppression intensifies the loss of consciousness to GAs (Fu et al., 2017). In this regard, Saalman and colleagues (Redinbaugh et al., 2020) recently demonstrated CL neurons in monkeys discharged at very high rates of activity in waking (40–50 Hz) and at significantly reduced rates during NREM sleep—as well as during general anesthesia. They further showed that CL stimulation in *anesthetized* monkeys rapidly restored arousal and consciousness. Tasserie et al. (2022) similarly found that DBS of the central, but not ventrolateral, thalamus in anesthetized non-human primates produced significant increases in cortical arousal as measured by EEG and fMRI—while also inducing “signatures of consciousness.”

Blumenfeld and colleagues (Feng et al., 2017; Kundishora et al., 2017; Xu et al., 2020) recently examined the role of CL in seizure activity. Gummadavelli et al. (2015) initially demonstrated that CL stimulation abolished slow wave activity during postictal periods in rats, together with the resumption of normal exploratory/motor behaviors. They subsequently reported that combined CL and the pontine RF stimulation in rats during focal seizures restored cortical arousal and behavioral responsiveness during both the ictal and postictal stages (Kundishora et al., 2017), and further that single CL stimulation activated the cortex and improved performance on an active avoidance task, postictally (Xu et al., 2020). Lastly, Martin et al. (2021) examined changes in EEG activity in epileptic patients following DBS stimulation of the intralaminar thalamus and noted progressive increases in gamma activity which corresponded to reduced alpha power, validating ILt enhancement of cortical arousal.

In summary, the foregoing indicates CL and CM of the rostral ILt are vital components of an extended circuitry which serves to maintain arousal and consciousness. Importantly, this neural network involving ILt, C-P and the cortex, by sustaining consciousness, may heighten arousal, to thereby, as Lin et al. (2016) stated “synchronize activity in neural networks that underlie cognition.”

#### ILt functional properties—Cognition

While the anterior (ATN) and midline nuclei of thalamus serve well-recognized roles in cognitive functions, the ILt nuclei also participate in higher order cognitive processes (for review, see Mair et al., 2011, 2021; Cover and Mathur, 2021). However, unlike the anterior and midline nuclei, which are strongly linked to the hippocampus and the PFC, ILt connections with corticostriatal circuits suggests an influence of cognition, mimicking that of the striatal circuitry. In this regard, studies which have compared the effects of ILt, ATN or midline thalamus on behavior have described distinct differences in cognitive processing among these nuclei. For instance, Mitchell and Dalrymple-Alford (2005, 2006) demonstrated that ATN lesions disrupted performance on a spatial radial arm maze (RAM) task, whereas ILt lesions produced impairments on a non-hippocampal-dependent (egocentric) working memory task. Consistent with this, Bailey and Mair (2005) demonstrated that ILt lesions did not alter performance on a delayed non-matching to sample RAM task, sensitive to ATN and hippocampal damage (Mair et al., 2003), but produced delay-independent impairments on an operant lever-pressing task, known to involve the sensorimotor cortex and striatum (Burk and Mair, 2001).

As discussed, Hembrook and Mair (2011) compared the effects of ILt or ventral midline thalamic (RE/RH) lesions on delayed non-match to sample RAM task, sensitive to hippocampal and PFC damage, and on a visuospatial reaction time (VSRT) task, responsive to striatal and dorsal frontal cortical alterations (Mair et al., 2002; Bailey and Mair, 2004). They reported a double dissociation: RE/RH lesions disrupted performance on the RAM but on not the VSRT task, while ILt lesions altered behavior on the VSRT task but not on the RAM task. The foregoing supports a direct role for the ILt in sensorimotor and instrumental WM tasks which recruit corticostriatal loops, but a lack of involvement in SWM and reference memory tasks associated with the hippocampus.

Kato et al. (2018) examined the effects of CL inputs to the dorsal striatum on sensory discrimination learning and behavioral flexibility in mice. Using immunotoxins to selectively destroy CL cells projecting to C-P, they showed that the loss of CL cells produced impairments in a two-choice visual discrimination reaction time task but did not alter performance on a spatial working memory task. They further showed that the chemogenetic inhibition of these CL cells disrupted the reversal learning and set shifting phases of a conditional visual discrimination task, and concluded that “CL thalamostriatal neurons play a key role in response selection and reaction time modulation during the performance phase of visual discrimination” (Kato et al., 2018).

ILt neurons synapse on cholinergic (ACh) interneurons of the C-P (Ding et al., 2010) to release ACh to the striatum (Consolo et al., 1996; Brown et al., 2010), and ACh cells, in turn, connect with and activate dopaminergic (DA) neurons to enhance the release of dopamine in the caudate-putamen (Ding et al., 2010; Threlfell et al., 2012). As such, the ILt may influence motor learning through the ACh-mediated efflux of DA to the striatum. In this regard, Cover et al. (2019) recently demonstrated that rostral ILt stimulation activated cholinergic (ACh) striatal neurons to release dopamine to the C-P, and further, using an optical intracranial self-stimulation paradigm, that ILt stimulation was rewarding as mice vigorously self-stimulated for it—an effect that was attenuated by blocking dopamine 1 receptors.

Finally, Wolff et al. (2022) recently examined the role of intralaminar-striatal projections using a task which requires rodents to make a complex motor response, necessitating temporal precision, in an instrumental conditioning task. Using a combination of lesion and pharmacogenetic manipulations, they found that the dorsolateral striatum (DLS), *via* projections to the motor cortex, was critical in the acquisition of this motor response. Interestingly, however, inhibition of the DLS had no effect on performance for animals already proficient in the task, but notably suppression of the intralaminar thalamic-striatal pathway significantly impaired the learned motor sequences—even in the well trained animals. According to the authors, this indicated that “DLS projecting thalamic neurons are essential not only for executing the learned skills but also for learning them, consistent with an important role for thalamostriatal synapses in the formation of the underlying memory” (Wolff et al., 2022). In summary, these studies highlight a key role for the rostral ILt, through connections with the striatum and cortex, across a host of sensorimotor, instrumental and cognitive functions.

## Comparisons of anatomical and functional properties of the midline thalamus (PV, RE) and the rostral intralaminar thalamus (CM, CL)

**Figure 9:**
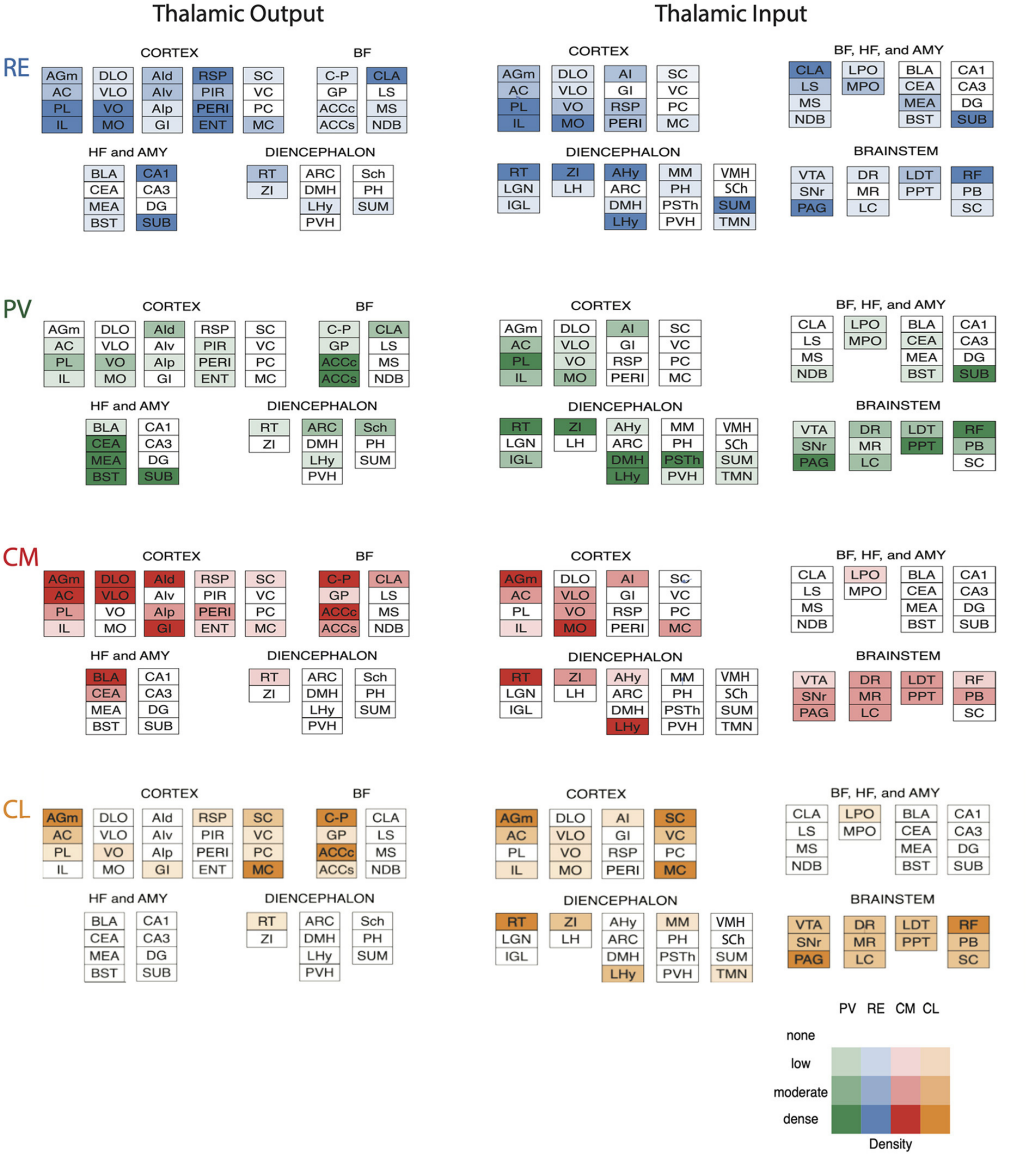
Schematic representation of the patterns and density of outputs (left) and inputs (right) of nucleus reuniens (RE) (blue) and the paraventricular (PV) nucleus (green) of the midline thalamus and the central medial (CM) (red) and central lateral (CL) (orange) nuclei of the rostral intralaminar thalamus. Note, while there are substantial differences in inputs/outputs from the cortex, striatum, and amygdala to the midline and rostral intralaminar nuclei, all nuclei receive strong (and overlapping) projections from brainstem “arousal-related” cell groups. Color coded density chart for input and outputs to each site at the bottom right. AC, anterior cingulate cortex; ACCc, nucleus accumbens core division; ACCs, nucleus accumbens shell division; AGm, medial agranular cortex; AHy, anterior hypothalamus; AI, agranular insular cortex; AId, dorsal insular cortex; AIp, posterior insular cortex; AIv, ventral insular cortex; AMY, amygdala; ARC, arcuate nucleus of hypothalamus; BF, basal forebrain; BLA, basolateral amygdala; BST, bed nucleus of the stria terminalis; CA, cornu ammonis; CEA, central nucleus of amygdala; CLA, claustrum; C-P, dorsal striatum; DG, dentate gyrus; DLO, dorsolateral orbital cortex; DMH, dorsomedial hypothalamus; DR, dorsal raphe nucleus; ENT, entorhinal cortex; GI, granular insular cortex; GP, globus pallidus; HF, hippocampus; IL, infralimbic cortex; IGL, intergeniculate leaflet of thalamus; LC, locus coeruleus; LDT, laterodorsal tegmental nucleus; LGN, lateral geniculate nucleus of thalamus; LH, lateral habenula; LHy, lateral hypothalamus; LS, lateral septum; LPO, lateral preoptic area; MEA, medial amygdala; MC, motor cortex; MO, medial orbital cortex; MPO, medial preoptic area; MM, mammillary nuclei of hypothalamus; MR, median raphe nucleus; MS, medial septum; NDB, nucleus of diagonal band; PAG, periaqueductal gray; PB, parabrachial nucleus; PC, parietal cortex; PERI, perirhinal cortex; PH, posterior hypothalamus; PIR, piriform cortex, PL, prelimbic cortex; PPT, pedunculopontine tegmental nucleus; PSTh, parasubthalamic nucleus; PVH, paraventricular hypothalamic nucleus; RSP, retrosplenial cortex; RF, pontomesencephalic reticular formation; RT, reticular nucleus of thalamus; SC, somatosensory cortex; Sch, suprachiasmatic nucleus; SNr, substantia nigra pars reticulata; SUB, subiculum; SUM, supramammillary nucleus of hypothalamus; TMN, tuberomammillary nucleus; VC, visual cortex; VLO, ventrolateral orbital cortex; VMH, ventromedial nucleus of hypothalamus; VO, ventral orbital cortex; VTA, ventral tegmental area; ZI, zona incerta.

As discussed, the anatomical and functional properties of the dorsal (PV) and ventral (RE) midline thalamus are strikingly different. In like manner, the anatomical and functional characteristics of ILt significantly differ from those of RE/PV. However, the functions of these thalamic nuclei complement one another, signifying an integrated role for the midline/ILt thalamus in limbic-associated functions. Figure 9 schematically compares the inputs and outputs of RE, PV, CM and CL, comparing densities and sites of projection across nuclei. Whereas, the rostral ILt share some inputs with RE/PV, the outputs from these two thalamic regions (RE/PV and ILt) largely diverge.

The midline and ILt nuclei receive a diverse array of input from the brainstem including aminergic and ACh nuclei, but the rostral ILt, distinct from PV/RE, receives prominent projections from the pontomesencephalic RF. In further contrast with RE/PV, the ILt receives only modest projections from the hypothalamus. Additionally, there are marked difference in cortical afferents to the midline and ILt thalamus. The sensorimotor cortex distributes densely to CL, while mainly avoiding midline structures. By comparison, the mPFC strongly targets the midline and intralaminar thalamus, with projections differing from the dorsal and ventral mPFC. For instance, the ventral mPFC (PL, IL) projects strongly to RE and PV, moderately to CM, and essentially avoids CL (Figures 2A–C). By contrast, the anterior cingulate cortex (AC) distributes heavily to CM, modestly to RE and CL, and sparsely to PV. Interestingly, ORB projections to the ILt show a medio-lateral gradient such that medial (MO/VO) divisions of ORB project more heavily to medial structures (CM), whereas lateral (VLO) divisions distribute more densely to lateral sites (CL).

With respect to output, t1he main targets of CL/PC are sensorimotor cortices and the dorsal striatum. CM also distributes heavily to these sites, but additionally to parts of the limbic cortex, to the ACC and to the amygdala, mainly to BLA. Unlike, however, PV and RE which are reciprocally linked with the HF/subiculum, there are no CM connections with the HF. In addition, in contrast to the absence of PV/RE projections to C-P, CM distributes massively throughout the dorsal striatum and modestly to ACC. Interestingly, CM lies along the midline, and as such shares projections with the dorsal and ventral midline thalamus; that is, limbic subcortical projections with PV and limbic cortical ones with RE. Accordingly, CM appears to serves as an anatomical and functional bridge to the dorsal and ventral midline thalamus.

**Figure 10:**
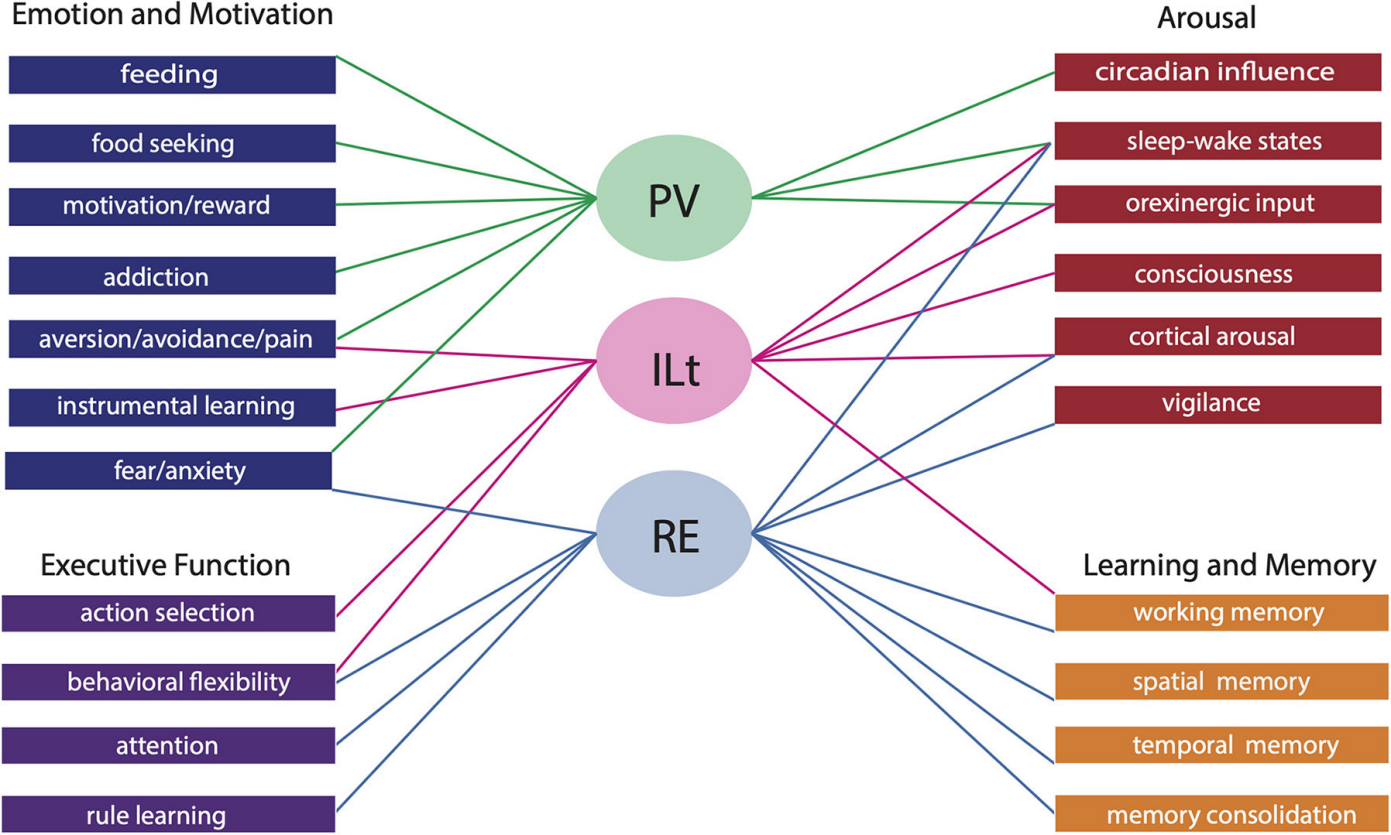
Schematic diagram illustrating the shared and unique functional contributions of the paraventricular nucleus (PV) of the dorsal midline thalamus, the nucleus reuniens (RE) of the ventral midline thalamus and the central medial, central lateral and paracentral nuclei of the rostral intralaminar thalamus (ILt). Both the midline and intralaminar nuclei participate in distinct roles in arousal, emotion, motivation and cognition. For instance, both PV and ILt have been linked to motivated behaviors, however PV plays a key role in feeding, appetitive and aversive conditioning and addiction while the ILt participates in instrumental conditioning and pain perception. By comparison, RE is involved in circuitry influencing innate and learned fear/anxiety. Similarly, the midline and intralaminar thalamus collectively drives arousal, however the ILt maintains consciousness while the midline nuclei receive hypothalamic and brainstem input which modulate states of arousal for effective responding—PV for circadian linked behaviors including feeding and RE for attentional/vigilant responding. Lastly, RE and the ILt contribute largely to cognition and both share a role in flexible goal directed behavior and working memory (WM), but each group inimitably subserve dissociable processes. RE is linked to attention in addition to the spatial and temporal components of WM/long-term memory while the ILt facilitates the sensorimotor components of WM.

Figure 10 illustrates various common and independent functions of the midline and intralaminar thalamic nuclei, reflecting their unique anatomical characteristics. Regarding functional properties, the ILt (or CL), has long been recognized as critical intermediary between the brainstem RF and the sensorimotor cortices in processes of arousal and consciousness. Perhaps unlike PV/RE, which transfer excitatory inputs from the brainstem/hypothalamus to the limbic forebrain to modulate states of arousal for effective responding (e.g., attention, feeding, motivation), the ILt appears critically important for maintaining consciousness, per se. Specifically, lesions/damage of the ILt, results in a loss of consciousness, whereas ILt/CL stimulation in rodents, monkeys or humans has been shown to restore consciousness from sleep or general anesthesia, or following thalamic damage.

Whereas, both RE and ILt subserve cognitive processes, their roles in cognition differ, undoubtedly owing to their differential projections: RE to limbic cortices and ILt/CL mainly to sensorimotor cortices and the dorsal striatum. Specifically, lesions/inactivation of the ILt produce impairments on cognitive tasks that involve sensorimotor components such as visuospatial, reaction time, instrumental, or sensory discrimination tasks, but essentially unlike RE, do not alter behavior on spatial working memory tasks.

## Conclusion

The midline and intralaminar nuclei of the thalamus have traditionally been characterized as a single or unified system with common projections and functions. As reviewed herein, there are not only marked anatomical and functional differences between the dorsal and ventral midline thalamus but also between the midline thalamus and the rostral intralaminar complex of the thalamus. While each of the midline and intralaminar nuclei perform distinct functions, they collectively serve a critical role in several affective, cognitive and executive behaviors—as major components of a limbic brainstem/diencephalic-thalamic-cortical circuitry (Figure 10).

## Data availability statement

The original contributions presented in the study are included in the article/supplementary material, further inquiries can be directed to the corresponding author.

## Author contributions

RV, SL, and AR contributed to writing. SL created the figures. All authors were involved in the preparation of the manuscript. All authors contributed to the article and approved the submitted version.

## Funding

This was supported by NIH grants: NS108259 and NS119847.

## Conflict of interest

The authors declare that the research was conducted in the absence of any commercial or financial relationships that could be construed as a potential conflict of interest.

## Publisher's note

All claims expressed in this article are solely those of the authors and do not necessarily represent those of their affiliated organizations, or those of the publisher, the editors and the reviewers. Any product that may be evaluated in this article, or claim that may be made by its manufacturer, is not guaranteed or endorsed by the publisher.
